# Income inequality and multimorbidity patterns in China: a micro-level analysis using CHARLS

**DOI:** 10.3389/fpubh.2025.1588325

**Published:** 2025-04-16

**Authors:** Mengqian Ouyang, Bowen Liu, Riping Xu

**Affiliations:** ^1^Department of Economics, Guangdong Institute of Public Administration, Guangzhou, China; ^2^Department of Biostatistics, Harvard T.H. Chan School of Public Health, Boston, MA, United States; ^3^Department of Anesthesiology, Affiliated Hospital of Guangdong Medical University, Zhanjiang, China

**Keywords:** multimorbidity, health inequality, relative income, Kakwani Index, CHARLS

## Abstract

**Introduction:**

Health inequality—particularly income-related health inequality—poses a global challenge, significantly affecting social and economic well-being. While previous research has investigated the link between income inequality and various health outcomes, including chronic diseases, studies focusing on multimorbidity remain limited.

**Methods:**

This study examines how income inequality affects multimorbidity in China, drawing on data from the China Health and Retirement Longitudinal Study. By employing the Kakwani Index, the analysis evaluates income inequality at the individual level and utilizes Latent Class Analysis to identify multimorbidity patterns. The research further explores how these effects vary across different age groups and regions. The study investigates the role of household economic decisions in shaping health outcomes. RIF regression is used to break down the contribution of income inequality to health disparities.

**Results:**

Lower relative wage income was strongly associated with an increased number of chronic diseases and heightened likelihood of specific multimorbidity patterns, particularly Respiratory-Cardiovascular diseases and overall disease burden. Redistribution of income partially alleviated the negative impact of income inequality on health outcomes. The effects of income inequality on health differ notably across age groups and geographical regions. Developmental expenditures (e.g., improving living conditions) were more effective in addressing income-related health disparities than direct increases in healthcare spending.

**Discussion:**

Policy responses need to focus on targeted income redistribution strategies and increased investment in developmental initiatives to address these growing health inequalities.

## 1 Introduction

Health inequality poses a significant challenge to contemporary society, profoundly impacting both social and economic domains. From a social perspective, it exacerbates group divisions. Disadvantaged populations are more likely to suffer from illnesses because they lack equal access to healthcare resources. This reduces their quality of life and may also lead to social tensions and threats to societal stability. From an economic perspective, health inequality undermines the quality and efficiency of the workforce, limits productivity growth, and hampers economic progress. While providing medical assistance to disadvantaged groups is essential, it may lead to increased healthcare expenditures, imposing long-term pressure on fiscal and social security systems. Therefore, health inequality is not only a core issue of public health but also deeply intertwined with social prosperity and sustainable economic development. Ignatow and Gutin argues that socioeconomic disparities adversely affect public health outcomes and they discuss how income inequality not only fuels health disparities but also states that improvements in health equity can bring about benefits such as enhanced societal well-being and reduced healthcare costs (1). Hurley found that people display a stronger aversion to income-related health inequalities than to income inequality or health inequality alone, providing evidence of the psychological stress caused by income disparities (2).

Health inequality arises from a complex interplay of multiple, interconnected factors. Gostin and Friedman point out that health outcomes are not solely determined by access to medical services but are also deeply influenced by structural social issues. Factors such as employment, education, housing, transportation, and racial discrimination are critical determinants of health. For instance, an infant born in an affluent white suburb of St. Louis, USA, has a life expectancy that is 35 years longer than one born just a few kilometers away in a low-income Black neighborhood (3). Existing research has systematically explored the mechanisms driving health inequality across various dimensions, including material resource distribution, social psychological factors, and individual behavior. From the perspective of material resource distribution, factors such as access to food, community environment, environmental pollution, and equal opportunities to obtain healthcare resources are closely related. Inequalities in the allocation of material resources among different groups directly lead to health disparities. From the perspective of social psychological factors, groups that experience higher levels of social exclusion and stress are more likely to develop psychological distress, which negatively affects their health through biological stress mechanisms. From the perspective of individual behavioral patterns, differences in health behaviors such as dietary habits, smoking rates, and participation in health check-ups across different groups further exacerbate health inequalities (4–7).

Income level is one of the key determinants of health inequality. Wilkinson and Pickett reviewed data from 168 studies across 155 publications, finding that ~70% of the analyses supported a negative correlation between income inequality and poorer health outcomes (8). Pickett and Wilkinson argue for a causal link between income inequality and adverse health outcomes, supported by established epidemiological criteria such as temporality and biological plausibility (9).

Researchers analyze income-related health inequality from various dimensions of health. Some studies focus on health risk factors, such as BMI, blood pressure, and waist circumference (10, 11); Others employ standardized health measurement tools, such as EQ-5D (12) 和 EQ-5D-5L (13, 14); Additionally, some scholars utilize individuals' self-rated health assessments (15, 16); In research on health inequality in low- and middle-income countries, 66.7% of studies define health inequality subgroups based on socioeconomic status, using measures such as wealth quintiles derived from household asset indices or regional classifications (17).

Scholars are increasingly investigating the relationship between income and chronic diseases. Sturm and Gresenz analyzed the link between income inequality and nine chronic diseases and found no significant association between income inequality and the prevalence of chronic diseases, either in the general population or among the poor (18); Qin et al. systematically examined the relationship between income levels and 14 types of chronic diseases, revealing that income has a substantial impact on the incidence of chronic diseases among individuals aged 45–59. Interestingly, the prevalence of heart disease was higher among wealthier individuals, which contrasts with trends observed in developed countries (19); Using cross-national data, Li et al. found that the burden of chronic obstructive pulmonary disease (COPD) is disproportionately concentrated among populations in countries with lower levels of socioeconomic development (20); He et al. constructed a composite health index using variables such as chronic disease status, self-rated health, and physician visits, discovering that income inequality negatively impacts overall individual health (21); Similarly, Li and Tang used chronic disease incidence as one of the indicators of health and found that chronic disease inequality, in relation to income, is pro-poor—indicating that poorer populations experience a higher inequality burden (16).

Multimorbidity significantly undermines individual health and places a heavy economic burden on individuals and families (22–24). As a result, researchers have begun focusing on the inequalities in multimorbidity associated with income. For example, Kunna et al. compared health inequalities from the perspective of wealth between China and Ghana. They found that in China, inequalities were particularly pronounced among the poor, with wealth quintiles contributing most to multimorbidity inequities. In Ghana, inequality was also significant but was more concentrated among the wealthy, with body mass index accounting for the largest contribution to multimorbidity inequality (25). Zhao et al. observed that physical multimorbidity was more prevalent in poorer regions compared to more affluent ones (26). Similarly, Mossadeghi et al. found that, among adults over 20 years old in the United States, living above the poverty level helped lower the likelihood of experiencing multimorbidity (27). La Porta and Zapperi examined a range of pathological conditions—such as cancer, diabetes, hypertension, heart disease, and obesity—and constructed multimorbidity matrices for individuals in the bottom and top quintiles of the income distribution. They identified clusters associated with hypertension, poor health, obesity, and diabetes among individuals in the lowest 20% income group in the United States (28). Additionally, Dugravot et al. measured socioeconomic status using education, occupational position, and literacy, and found that lower socioeconomic status significantly promoted the transition from healthier conditions to multimorbidity. However, it did not have a significant impact on the transition from multimorbidity to death (29). Despite these findings, the existing literature on inequalities in multimorbidity remains relatively limited. Many studies focus solely on the number of chronic conditions, leaving substantial room for further analysis of the patterns of multimorbidity across multiple chronic diseases.

This study assesses health status by measuring the number of chronic diseases and patterns of multimorbidity, using the Kakwani Index to determine income's relative position within social groups. It examines income-related health inequality, its heterogeneity across age groups and regions, and explores how raising average income levels and household economic decision-making might influence such health inequality. The contributions of this paper are as follows.

First, this study employs the Kakwani Index to measure income inequality among individuals, offering a distinct approach compared to conventional income stratification methods. The Kakwani Index not only represents income disparities more precisely—reflecting psychological stress and access to social resources—but also facilitates an analysis of how income inequality affects individual health, surpassing macro-level measures like regional income disparities.

Second, this study extends health inequality research into the field of multimorbidity. Given the increasing prevalence of multimorbidity and its substantial economic burden on national healthcare systems, investigating multimorbidity offers more practical and actionable insights compared to studying single chronic disease inequalities.

Third, this study provides policy suggestions by exploring solutions to income-related health inequality at both the micro and macro levels. At the micro level, it examines how household health decision-making influences such inequality, while at the macro level, it discusses whether raising aggregate income levels across the population can help alleviate it. These insights aim to inform policymakers with more nuanced recommendations.

Fourth, considering the specific context of China, where most individual income is derived from wage earnings—a primary economic source for health—this study accounts for three income types: personal wage income, post-transfer income, and family-supported income. Transfer income is a crucial tool used by the government to address income disparities, while traditional family-oriented culture in China means that income from other family members plays a significant role in mitigating financial risks related to health. Analyzing these three income types enables the study to elucidate the roles of individuals, governments, and families in supporting individual health.

## 2 Methods

### 2.1 Data source and study sample

The data used in this study are sourced from the publicly available dataset of the China Health and Retirement Longitudinal Study (CHARLS), which can be accessed through the official project website (https://charls.pku.edu.cn/).

The China Health and Retirement Longitudinal Study (CHARLS) is a nationally representative longitudinal survey that began with a baseline conducted in 2011–2012, covering 17,708 individuals from 10,257 households across 28 provinces in China using a multistage probability proportional to size (PPS) sampling method with implicit stratification by region, urban/rural classification, and per capita GDP, offering a comprehensive and representative microdataset on households and individuals. The survey's comprehensive questionnaire captures data on demographics, family support, health, healthcare utilization, work, retirement, income, and community conditions, maintaining national and regional representativeness despite challenges such as COVID-19-related disruptions in 2020.

The data from 2013 and later are closer to the current socio-economic context and offer greater consistency in survey questions. Therefore, this paper selects data from the 2nd, 3rd, 4th, and 5th waves of the CHARLS database, namely the data from 2013, 2015, 2018, and 2020.

The data cleaning and organization process involved several steps. First, datasets from the modules of basic information, household information, health status and functioning, work and retirement, and income and expenditure were merged. Subsequently, samples with missing income or chronic disease data were excluded, as they were deemed unsuitable for analysis. Then, we applied a 1% lower-tail trimming for individual wage income, individual transfer income, and household income from other family member and performed 1% lower- and upper-tail trimming for household agricultural business income and household non-agricultural business income. Missing values were then handled through imputation: the sample mean was used to replace missing values for continuous variables, while the sample mode was applied to impute missing values for discrete variables.

After completing these steps, a total of 67,866 valid samples were obtained. Of these, 14,160 samples were from 2013, 14,247 from 2015, 19,679 from 2018, and 19,330 from 2020.

### 2.2 Dependent variables

The dependent variable is health status, represented by the presence of multimorbidity. The study includes 14 chronic diseases: hypertension, dyslipidemia, diabetes, malignant tumors, chronic lung diseases, liver diseases, heart disease, stroke, kidney diseases, gastrointestinal disorders, mental health conditions, memory impairments, arthritis, and asthma. Multimorbidity is measured using two approaches: the number of chronic diseases and the patterns of chronic diseases that occur simultaneously. Latent Class Analysis (LCA) was used to classify the chronic disease data into distinct patterns, with each category denoting a unique multimorbidity pattern.

### 2.3 Independent variables

The independent variable examined in this study is the respondents' disposable income. According to income sources, the National Bureau of Statistics of China classifies disposable income into four categories: wage income, net operating income, net property income, and net transfer income. According to the China Statistical Yearbook, during the four survey years examined in this study, the national per capita wage income share was 56.9%, 56.7%, 56.1%, and 55.7%, respectively; the share of net operating income was 18.8%, 18.0%, 17.2%, and 16.5%; the share of net property income was 7.8%, 7.9%, 8.4%, and 8.7%; and the share of net transfer income was 16.6%, 17.4%, 18.3%, and 19.2%.

These data indicate that most disposable income in China originates from wage income, which includes earnings from various forms of labor compensation and benefits. This is followed by transfer income, such as pensions, poverty alleviation subsidies, and employment subsidies received by individuals after retirement. Net operating income refers to the net income generated by households or household members from production and business activities, usually taking the family as a whole as the production unit. In the survey, this type of income is further divided into income from agricultural and non-agricultural business activities. These incomes can be either positive or negative. Additionally, some individuals may have no income of their own but can receive financial support from other family members.

Accordingly, based on the practical context of the study and the design of the survey questionnaires, this paper selects individual wage income, individual transfer income, income from other family members, household agricultural net operating income, and household non-agricultural net operating income as sources of income, all of which contribute to the costs of preventing and treating chronic diseases.

When considering an individual's relative position in income distribution within the population, the study takes a cumulative perspective to analyze three dimensions of income exploitation as the main independent variables. The first is wage income alone. The second is total income after transfers, calculated as the sum of wage income and transfer income. The third is income supported by the family, calculated as the sum of wage income, transfer income, and income from other family members. The latter two variables allow for a clearer assessment of the redistributive role of government policies and the risk-sharing role of families. The traditional concept of filial piety, in which children are expected to care for their parents in old age, is deeply entrenched in Chinese culture, highlighting the significant role children play in mitigating health-related financial shocks for their parents.

Furthermore, the range of household agricultural operating income and non-agricultural operating income is (–∞, +∞). Specifically, a negative value indicates a household operating loss, a positive value indicates a household operating profit, and a value of zero indicates that the household's operations are at breakeven. The negative values of household agricultural and non-agricultural operating incomes may significantly alter the shape of the Lorenz curve and even result in “reversals,” thereby affecting the calculation of the Kakwani index. Therefore, these two income variables are included separately as independent variables in the model, rather than calculating the Kakwani index for the income that includes them.

### 2.4 Confounding factor

The study's control variables are organized into four categories:

1. Lagged chronic disease variables: Chronic diseases tend to develop slowly, have long durations, and are difficult to cure completely. However, with proactive treatment, their progression can be controlled. Given that this study leverages panel data, the number of chronic diseases or multimorbidity types from the previous survey wave is included as an independent variable to account for the persistence of chronic diseases.
2. Individual characteristics: These include occupation, education level, gender, age, household registration type, marital status, smoking status, and alcohol consumption frequency. Specifically, occupation is represented by five dummy variables indicating whether an individual is (i) self-employed in agriculture, (ii) employed in government departments, public institutions, or non-profit organizations, (iii) employed by enterprises, (iv) self-employed as a business owner, or (v) a farmer. Education is treated as an ordinal variable, ranging from 1 to 11, corresponding to increasing levels of education: illiteracy, dropped out of primary school, private school, primary school, junior high school, senior high school, vocational school, junior college, undergraduate, master's degree, and doctoral degree.
3. Regional fixed effects: The sample is divided into four regions—eastern, central, western, and northeastern—following the classification standards of the China National Bureau of Statistics.
4. Time fixed effects: Dummy variables based on survey years are included to control for temporal heterogeneity.

### 2.5 Descriptive statistics of variables

**Figure 1:**
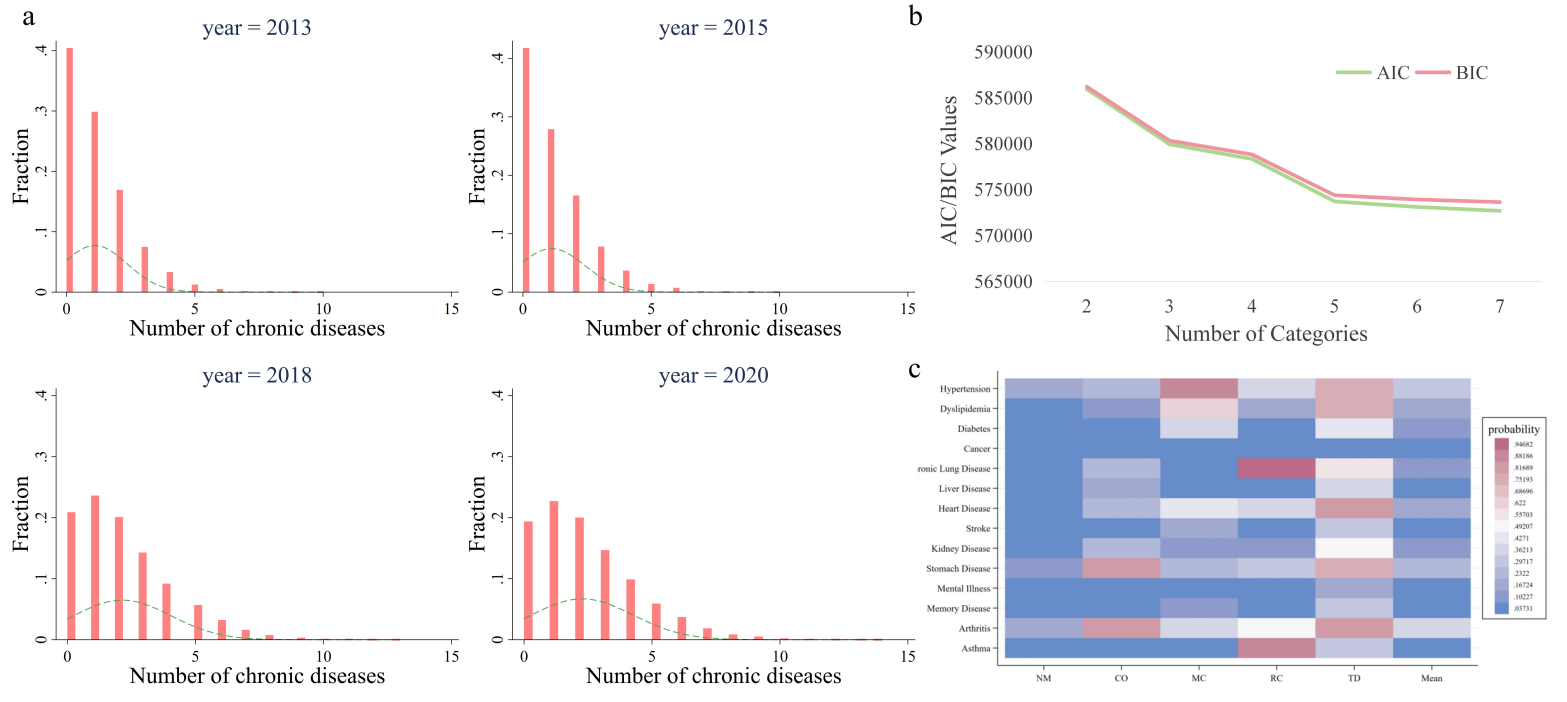
Quantity and patterns of multimorbidity. **(a)** Shows the histogram of quantity of multimorbidity. The height of the bars indicates the proportion of the corresponding sample size to the total sample, while the dashed line depicts the density curve. **(b)** Shows the selection process of the number of classes in Latent Class Analysis. AIC (Akaike Information Criterion) measures the trade-off between model fit and complexity, with lower values indicating a better model. BIC (Bayesian Information Criterion) extends this by introducing a stronger penalty for model complexity, favoring simpler models with fewer parameters. **(c)** Illustrates multimorbidity patterns. The *y-*axis shows 14 chronic diseases, the *x-*axis displays 5 multimorbidity classes, and the color of each cell reflects the conditional probability of having a specific chronic disease within a given comorbidity class. A deeper red indicates a higher probability of having the chronic disease. NM, CO, MC, RC, and TD represent No Multimorbidity, Complex-Organ diseases, Metabolic-Circulatory diseases, Respiratory-Cardiovascular diseases, and Total diseases, respectively.

In Figure 1a, we presents histograms illustrating the distribution of the number of chronic diseases across different survey waves. First, in 2013, ~40% of individuals were not diagnosed with any chronic diseases; this proportion increased slightly to over 40% in 2015 but subsequently declined to around 20% in 2018 and 2020, indicating a rising prevalence of chronic diseases over time. Second, the proportion of individuals with only one chronic disease decreased from 30% in 2013 to 22% in 2020, suggesting a reduction in cases with just a single chronic condition. Third, the overall shape of the histograms shows a declining trend in the proportion of individuals as the number of chronic diseases increased in 2013 and 2015. However, this trend shifted to an asymmetric inverted U-shape in 2018 and 2020, with a thickening tail on the right-hand side. This suggests a growing number of individuals with multiple chronic diseases, possibly due to inadequate treatment or rest, leading to the progression from a single condition to multimorbidity. Overall, there is a clear trend of Chinese residents transitioning from having no or a single disease to multiple chronic conditions, which is likely to pose significant future burdens on medical insurance funds and households.

Among individuals without any chronic diseases, 71.37% had wage incomes below 100 yuan, and 77.49% belonged to households whose other members had a total income ≤0 yuan. As the number of chronic diseases increased, the proportion of individuals with wage incomes below 100 yuan gradually rose, as did the proportion of households with other members earning ≤0 yuan. This indicates a correlation between the number of chronic diseases and lower levels of personal income and poorer household economic conditions. On the other hand, as the number of chronic diseases increased from none to four or more, the proportion of individuals with transfer incomes exceeding 1,000 yuan grew from 7.29% to 19.54%. This highlights the potential role of transfer income in alleviating the financial burden of medical expenses for individuals and families. Descriptive statistics for other variables can be found in Supplementary Table A.1.

### 2.6 Statistical analysis

#### 2.6.1 Identifying patterns of multimorbidity using latent class analysis

Hypertension, gastrointestinal disorders, and Alzheimer's disease are the most prevalent conditions, regardless of whether individuals have single or multiple diseases. However, as the number of chronic diseases increases, the prevalence of dyslipidemia and cardiovascular diseases rises sharply. This indicates that if existing chronic diseases are not effectively managed, individuals may become more prone to developing conditions related to lipid metabolism and heart health. We used Stata's gsem command, setting the seed number to 10,000 and using default parameters, to compute the LCA results for 1–7 classes. The corresponding AIC (Akaike Information Criterion) and BIC (Bayesian Information Criterion) are shown in Supplementary Figure A.1.

Nonetheless, this preliminary analysis does not fully capture the interrelationships among the 14 chronic diseases. To address this, we utilized Latent Class Analysis (LCA) to identify multimorbidity patterns among these diseases (30). Based on the fundamental assumption of LCA, chronic diseases are considered external manifestations of an individual's underlying health status. Individuals in different latent health states exhibit similarities in the presence of certain chronic conditions. These latent states can be modeled using a limited number of mutually exclusive categorical variables that explain the probability distribution of the 14 chronic diseases, allowing us to classify individuals into distinct multimorbidity patterns (31). LCA has notable advantages. Unlike the K-means algorithm, it is not sensitive to the order of the input data. Additionally, it makes full use of all the data, which provides an edge over principal component analysis.

#### 2.6.2 Analyzing how the relative income position affects health outcomes

First, we calculate the Kakwani index to assess the relative degree of income exploitation at an individual level (32), reflecting an individual's position within their group. The index ranges from [0, 1], where a higher value indicates a greater degree of income exploitation, higher psychological stress, and fewer social resources available to the individual (33–35). This measurement approach is widely used in the literature to measure the income inequality (36–38). The calculation steps for the Kakwani index are as follows:

1. In a specific subgroup with a total sample size of *n*, arrange individual incomes in ascending order to obtain an income sequence(*y*_1_, *y*_2_, ..., *y*_*n*_).
2. Calculate the sample's mean income μ_*Y*_. For individuals in the sequence exceeding income level *y*_*k*_, compute the proportion of such individuals $\gamma_{y_{k}}^{*}$ and their average income $\mu_{y_{k}}^{*}$.
3. Use these values to compute the Kakwani index $R\left({y,y}_{k}\right)=\gamma_{y_{k}}^{*}\left(\mu_{y_{k}}^{*}-y_{k}\;\right)/\mu_{Y}$.

Next, we calculate the Kakwani index for three income levels:

1. Personal wage income: This reflects an individual's wage income only.
2. Post-transfer income: This includes both personal wage income and transfer income, adjusted to reflect income levels after government redistribution.
3. Family-supported income: This aggregates personal wage income, transfer income, and income from other family members.

The Kakwani index offers a versatile and robust approach to income distribution analysis by capturing both relative advantage and deprivation, remaining unaffected by income scale fluctuations, being applicable across various levels and contexts, and providing unique insights into the relative positions and subjective perceptions of inequality often overlooked by traditional methods.

Finally, panel Tobit and Logit models are employed to estimate the effects of the three Kakwani indices on the number of chronic diseases and the occurrence of specific comorbidities. These models allow us to explore how an individual's relative income position within a group affects their health outcomes. The model specifications are provided in Equations 1, 2.


(1) $$\begin{array}{c} chronic_{it}^{*}=\alpha_{0}+\alpha_{1}chronic_{i,t-1}+\beta_{1}Kakwani_{it}\times income_{it}^{\prime }\beta_{2}\\ +control_{it}^{\prime }\gamma +u_{i}+\varepsilon_{it}\\ chronic_{it}^{*}=14\;if\;chronic_{it}^{*}>14\\ chronic_{it}^{*}=0\;if\;chronic_{it}^{*}<0 \end{array}$$

(2) $$\begin{array}{l} \;P\left(cluster_{it}=1|cluster_{i,t-1},Kakwani_{it},income_{it},control_{it}\;\right)=\\ \Phi \left(\alpha_{0}+\alpha_{1}cluster_{i,t-1}+\beta_{1}Kakwani_{it}+income_{it}^{\prime }\beta_{2}+control_{it}^{\prime }\gamma \right) \end{array}$$

In order to control for unobservable individual fixed effects, the choice of control variables varies depending on the specific income measure used to calculate the Kakwani index. When the Kakwani index is derived from personal wage income, *income*_*it*_ include transfer income, income from other family members, household agricultural operating income, and household non-agricultural operating income. When the Kakwani index is calculated using post-transfer income, *income*_*it*_ are income from other family members, household agricultural operating income, and household non-agricultural operating income. Finally, when the Kakwani index is based on family-supported income, *income*_*it*_ are limited to household agricultural operating income and household non-agricultural operating income.

#### 2.6.3 Exploring the heterogeneity of impact of relative income position on health outcomes

From the perspective of individual age, the incidence of chronic diseases is closely related to age. As individuals grow older, their physical functions gradually deteriorate, leading to a significant increase in the prevalence of multimorbidity. Among patients aged 65 and above, there is a strong correlation between several conditions, including cerebrovascular diseases, heart disease, lipoprotein metabolism disorders, and peripheral vascular diseases (39). Older adults often face significant challenges in engaging with complex tasks or physically demanding labor. Consequently, their relative income positions within the group are more likely to be influenced by objective physical health conditions rather than by psychosocial mechanisms that impact health outcomes. To better understand the differential effects of the relative position of income on health across age groups, we divided the sample into two subgroups using 65 years as the age threshold and conducted a comparative analysis.

From the perspective of social environments, China exhibits pronounced regional economic disparities. For example, in 2023, Shanghai, located in the eastern region, had a per capita GDP of 190,321 yuan, comparable to the levels of Portugal, a developed country. Meanwhile, Gansu Province in the western region had a per capita GDP of 47,867 yuan, similar to that of Belarus. Due to these disparities, residents in eastern and western regions display different tendencies when balancing health and income. In the economically advanced eastern regions, where life moves at a faster pace and social pressures are greater, people are more inclined to prioritize income generation to maintain their health. In contrast, the western regions, which do not experience the same level of intense competition, may exhibit a greater tendency to protect health by moderating effort levels.

Moreover, the urban-rural dual structure is a key characteristic of developing countries. Social policies implemented by the government in urban and rural areas, such as medical insurance and pension schemes, often differ in terms of coverage and subsidy standards. As the largest developing country in the world, China still faces significant disparities between urban and rural residents in income, consumption, and access to social security resources. These disparities influence the health outcomes of urban and rural residents through channels such as resource allocation, consumption behavior, and social order. This paper conducts separate analyses of urban and rural areas to more accurately reveal the mechanisms through which income inequality affects various chronic diseases in different regions.

Thus, we classified the sample into eastern and western subgroups or urban and rural subgroups based on individuals' place of residence to facilitate further stratified analysis.

#### 2.6.4 Discussing the moderating effect of household economic decisions on health inequality

While chronic diseases are typically long-term, difficult to cure, and often persist over a lifetime, individuals can take proactive measures to manage their health. Actions such as maintaining a balanced diet, engaging in regular exercise, and adopting healthy lifestyle habits can help reduce the risk of developing chronic diseases or prevent them from progressing to multimorbidity.

In this study, we examine the moderating effect of two categories of household expenditures in health inequality. The first is developmental expenditure, which includes spending on healthcare, education and training, durable goods, cars, and communication and transportation devices, excluding medical expenses. The second is health expenditure, covering costs associated with fitness activities, sports equipment, and health products such as nutritional supplements. For both categories, we calculate their respective proportions of total household expenditure.

In our model, we construct interaction terms by combining the expenditure proportions from the previous period with current income variables to assess whether past health-related economic decisions can mitigate health inequality. The specific model is presented in Equation 3, with variable definitions identical to those in Equation 1.


(3) $$\begin{array}{c} chronic_{it}^{*}=\alpha_{0}+\alpha_{1}chronic_{i,t-1}+\beta_{1}Kakwani_{it}\times expenditure_{it}\\ +income_{it}^{\prime }\beta_{2}+control_{it}^{\prime }\gamma +u_{i}+\varepsilon_{it}\\ chronic_{it}^{*}=14\;if\;chronic_{it}^{*}>14\\ chronic_{it}^{*}=0\;if\;chronic_{it}^{*}<0 \end{array}$$

#### 2.6.5 Decomposing health inequality using RIF regression

From the perspective of the number of chronic conditions, three metrics—Concentration Index, Gini Coefficient, and Quantile Differences—were employed to measure the degree of health inequality. The Concentration Index was calculated based on the ranking of total income, which includes individual wages, individual transfer income, total income from other household members, household agricultural income, and household non-agricultural income. Quantile Differences, on the other hand, were measured as the differences between the 90th and 10th percentiles, the 80th and 20th percentiles, and the 70th and 30th percentiles. These quantile-based measures provided a more precise decomposition of health inequality. However, it is important to note that while quantile differences capture the polarization in the number of chronic conditions, they do not indicate whether chronic conditions are concentrated at lower or higher levels. Therefore, these results should be interpreted in conjunction with other metrics. In terms of multimorbidity patterns, where all variables are binary, the Erreygers Concentration Index (EI), an adjusted concentration measure, was employed to evaluate health inequality (40).

For the two dimensions of health inequality, namely the number of chronic diseases and multimorbidity patterns, RIF (Recentered Influence Function) regression was employed to decompose the effects across different income levels and other factors (41). RIF incorporates health distribution characteristics into a standard regression framework, enabling researchers to intuitively analyze how various factors influence changes in the characteristics of health distribution, rather than being limited to mean-based analysis. This approach enhances its explanatory power and applicability in the study of inequality. The RIF model is presented in Equation 4.


(4) $$\begin{array}{l} RIF\left\{chronic_{it},\upsilon \left(F_{chronic}\right)\right\}=\alpha_{0}+\alpha_{1}chronic_{i,t-1}+income_{it}^{\prime }\beta \\ \;+control_{it}^{\prime }\gamma +\varepsilon_{it} \end{array}$$

We focus on comparing the contributions of various income sources to health inequality, including individual wage income, individual transfer income, total income from other family members, household agricultural income, and household non-agricultural income.

These analyses was performed using Stata version 15.

## 3 Results

### 3.1 Latent class analysis of multimorbidity patterns

In the Latent Class Analysis, we tested models with 2, 3, 4, 5, 6, and 7 classes. AIC (Akaike Information Criterion) and BIC (Bayesian Information Criterion) steadily decreased as the number of classes increased, with a noticeable inflection point at 5 classes (Figure 1b). Therefore, we conducted the Latent Class Analysis using 5 classes.

The results of the latent class analysis identifying multimorbidity patterns are presented in the form of a heatmap (Figure 1c). Five distinct patterns shows specific prevalence trends across 14 chronic diseases:

1. No Multimorbidity (NM): Characterized by lower-than-average prevalence rates of all chronic diseases across the sample.
2. Complex-Organ diseases (CO): Exhibited below-average prevalence rates for malignancies, chronic lung diseases, liver diseases, heart diseases, kidney diseases, gastrointestinal or digestive disorders, mental illnesses, and arthritis. This pattern is primarily associated with diseases involving organ-specific dysfunction.
3. Metabolic-Circulatory diseases (MC): Showed below-average prevalence rates for hypertension, dyslipidemia, diabetes, malignancies, heart diseases, stroke, kidney diseases, memory-related disorders, and arthritis. Many of these conditions are frequently referred to as “diseases of affluence” in colloquial terms.
4. Respiratory-Cardiovascular diseases (RC): Displayed lower-than-average prevalence of hypertension, chronic lung diseases, heart diseases, kidney diseases, gastrointestinal or digestive disorders, mental illnesses, arthritis, and asthma.
5. Total diseases (TD): Demonstrated higher-than-average prevalence rates for all chronic diseases studied.

### 3.2 The impact of relative income position on multimorbidity

**Figure 2:**
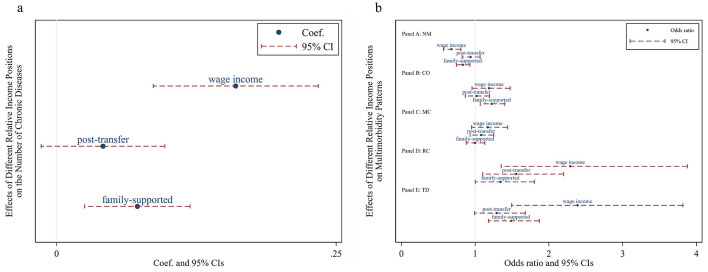
Effects of relative income position on multimorbidity. *Wage income* represents the relative position of personal wage income within the group; *post-transfer* represents the relative position of post-transfer income within the group; *family-supported* represents the relative position of family-supported income within the group. NM, CO, MC, RC, and TD represent No Multimorbidity, Complex-Organ diseases, Metabolic-Circulatory diseases, Respiratory-Cardiovascular diseases, and Total diseases, respectively. **(a)** Presents the results of the panel Tobit model, while **(b)** displays the results of the panel Logit model in the form of odds ratios.

The regression coefficient of the Kakwani index based on individual wage income is positive, with a 95% confidence interval to the right of zero, indicating a significant positive impact on the number of chronic diseases. The lower the individual's relative wage income position within the group, the more chronic diseases they tend to have, highlighting a significant pro-poor health inequality in this dimension. The Kakwani index based on post-transfer income has no significant effect on the number of chronic diseases, suggesting that income redistribution can mitigate the pro-poor tendency of chronic disease incidence. In contrast, the Kakwani index under household expenditure shows a significant positive impact on the number of chronic diseases, albeit to a lesser extent than the Kakwani index based on individual wage income. This indicates that support from other family members can exacerbate the inequality in the number of chronic diseases (Figure 2a).

According to the Kakwani index based on individual wage income, a lower relative position increases the likelihood of having Respiratory and Cardiovascular Disorders and total diseases, while decreasing the likelihood of having no multimorbidity. The Kakwani index based on post-transfer income indicates that a lower relative position increases the risk of Respiratory and Cardiovascular Disorders but has no significant effect on other multimorbidity patterns. Finally, the Kakwani index under household support shows a significant association between low income and higher prevalence rates of Complex Organ and total diseases. Notably, none of the three Kakwani indices have a significant impact on the Metabolic-Circulatory pattern, suggesting that this multimorbidity pattern may be prevalent among both the poor and the wealthy (Figure 2b).

### 3.3 The age heterogeneous effects of relative income position on multimorbidity

**Figure 3:**
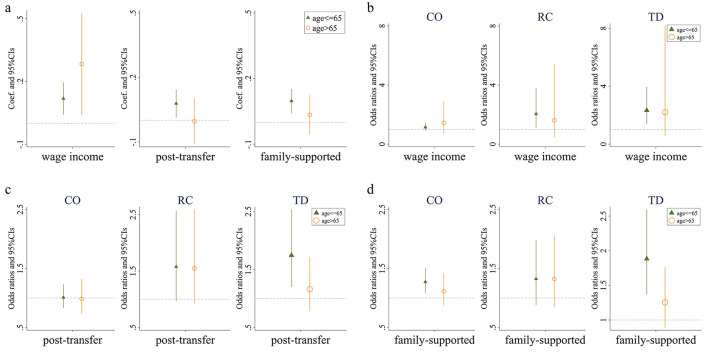
Effects of relative income position on multimorbidity. **(a)** Presents the impact of three types of income on the number of chronic diseases among individuals of different ages. **(b)** Presents the effect of wage income on the prevalence of Complex-Organ diseases, Respiratory-Cardiovascular diseases, and Total diseases across various age groups. **(c)** Presents the influence of post-transfer income on the incidence of these diseases among different age cohorts. Finally, **(d)** presents how income supported by family affects the prevalence of Complex-Organ diseases, Respiratory-Cardiovascular diseases, and Total diseases in individuals of varying ages. *Wage income* represents the relative position of personal wage income within the group; *post-transfer* represents the relative position of post-transfer income within the group; *family-supported* represents the relative position of family-supported income within the group. CO, RC, and TD represent Complex-Organ diseases, Respiratory-Cardiovascular diseases, and Total diseases, respectively. The coefficients are presented with 95% confidence intervals.

Whether in the group aged 65 and below or the group aged above 65, the Kakwani index of individual wage income demonstrates a positive effect on the number of chronic diseases. This indicates that individuals with a lower relative wage income position within their group are more likely to suffer from a higher number of chronic diseases, consistent with the main regression results. On the other hand, the Kakwani index of post-transfer income exhibits a positive effect on the number of chronic diseases for individuals aged 65 and below, suggesting that lower relative post-transfer income increases the likelihood of having chronic diseases or a greater number of chronic diseases within this group. However, this effect is not evident among individuals aged above 65. Similarly, the Kakwani index of family support income shows a comparable pattern in the regression results. These findings imply that income redistribution can effectively alleviate income-related health inequalities, particularly for the population aged above 65 (Figure 3a).

We then replaced the dependent variable with whether individuals belong to specific multimorbidity patterns and repeated the analysis. First, regardless of the Kakwani index used, the relative income position has no significant effect on the Complex organ and Metabolic-Circulatory diseases in either age group. Second, for the Respiratory and Cardiovascular pattern and the total diseases pattern, the Kakwani index of individual wage income shows a positive effect in the group aged 65 and below, but no impact in the group aged above 65. The Kakwani index of post-transfer income and family support income demonstrates no significant effect on the respiratory-cardiovascular diseases pattern in either age group, but retains a positive effect only for the total diseases pattern in the group aged 65 and below. Lastly, regarding the absence of chronic diseases, the likelihood of having no chronic diseases among individuals aged 65 and below is significantly influenced by their relative position in terms of wage income and family support income, while for individuals aged above 65, the absence of chronic diseases is not affected by any form of relative income position (Figures 3b–d; Supplementary Figure A.2).

### 3.4 The regional heterogeneity of the impact of relative income position on multimorbidity

Consistent with the main regression results, individuals with lower relative positions based on wage incomes tend to suffer from a greater number of chronic conditions, regardless of whether they live in eastern or non-eastern regions. However, there is no significant relationship between relative positions based on transfer income and the number of chronic conditions. When considering income supported by family contributions, a lower relative position is associated with poorer control of multimorbidity in eastern regions, whereas this relationship is not observed in non-eastern regions. Notably, across all measures of the Kakwani index, household agricultural income shows a significant negative effect on the number of chronic conditions exclusively in non-eastern regions, suggesting that increasing agricultural income helps reduce the prevalence of multimorbidity in these regions. This finding reflects the greater reliance on agriculture for livelihoods in non-eastern areas (Table 1).

**Table 1:** Regional heterogeneity of the effect of relative income position on number of multimorbidity.

**Independent**	**(I)**	**(II)**	**(III)**	**(IV)**	**(V)**	**(VI)**
**Variables**	**East**	**Not East**	**East**	**Not East**	**East**	**Not East**
*Wage income*	0.1622^**^ [0.0471, 0.2772]	0.1487^**^ [0.0527, 0.2447]				
*Post-transfer*			0.0452 [−0.0440, 0.1343]	0.0328 [−0.0377, 0.1032]		
*Family-supported*					0.0833^*^ [0.0071, 0.1595]	0.0590 [−0.0008, 0.1187]
Transfer income	0.0101 [−0.0132, 0.0334]	0.0133 [−0.0049, 0.0315]				
Other family members' income	−0.0082 [−0.0179, 0.0015]	−0.0064 [−0.0149, 0.0021]	−0.0083 [−0.0180, 0.0014]	−0.0065 [−0.0150, 0.0020]		
Family agricultural operating income	−0.0085 [−0.0322, 0.0151]	−0.0153^*^ [−0.0300, −0.0006]	−0.0080 [−0.0316, 0.0156]	−0.0155^*^ [−0.0302, −0.0008]	−0.0079 [−0.0316, 0.0157]	−0.0155^*^ [−0.0302, −0.0008]
Family non-agricultural operating income	−0.0085 [−0.0189, 0.0019]	−0.0001 [−0.0088, 0.0086]	−0.0083 [−0.0187, 0.0020]	−0.0004 [−0.0091, 0.0083]	−0.0085 [−0.0189, 0.0019]	−0.0005 [−0.0092, 0.0082]

Due to space limitations, lagged dependent variables and control variables are omitted; ^*^p < 0.05, ^**^p < 0.01; Confidence intervals are in parentheses. Variables related to absolute income level are divided by 10,000. *Wage income* represents the relative position of personal wage income within the group; post-transfer represents the relative position of *post-transfer* income within the group; *family-supported* represents the relative position of family-supported income within the group.

**Figure 4:**
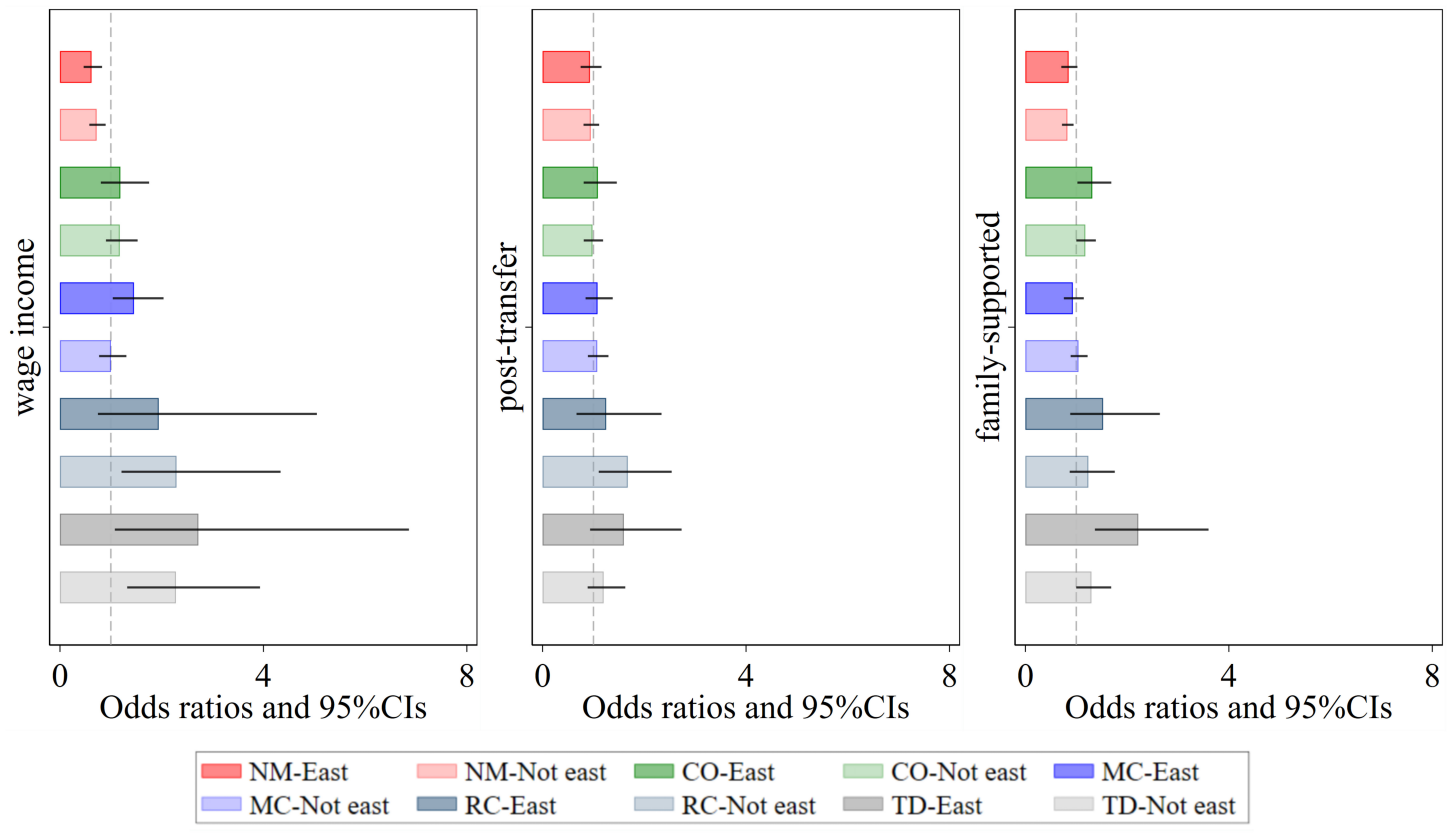
Regional heterogeneity of the effect of relative income position on patterns of multimorbidity. Darker shades represent the eastern region, while lighter shades represent the non-eastern region. *Wage income* represents the relative position of personal wage income within the group; *post-transfer* represents the relative position of post-transfer income within the group; *family-supported* represents the relative position of family-supported income within the group. NM, CO, MC, RC, and TD represent No Multimorbidity, Complex-Organ diseases, Metabolic-Circulatory diseases, Respiratory-Cardiovascular diseases, and Total diseases, respectively. The coefficients display the 95% confidence intervals. Results of the panel Logit model are presented in the form of odds ratios.

Hazard ratios (HRs) associated with the impact of income exploitation indices on the number of chronic conditions are shown (Figure 4). First, regional differences in the effects of personal income exploitation on various multimorbidity patterns are most pronounced for Metabolic-Circulatory diseases, Respiratory and Cardiovascular diseases, and total diseases. Specifically, the promotive effect on Metabolic-Circulatory diseases and total diseases is stronger and more significant in eastern regions, whereas the promotive effect on Respiratory and Cardiovascular diseases is greater and more distinct in non-eastern regions. Second, after income redistribution adjustments, personal income does not exhibit any significant effect on multimorbidity patterns, with the exception of a notable positive effect on Respiratory and Cardiovascular diseases in non-eastern regions. Third, higher levels of personal and household income exploitation indices are associated with an increased likelihood of total diseases, with the effect being stronger in eastern regions than in non-eastern regions. Additionally, the likelihood of Complex organ diseases rises as the personal and household income exploitation indices increase, although the differences between eastern and non-eastern regions are relatively small.

Based on the types of household registration, the sample was categorized into urban and rural subgroups for comparative analysis. As shown in Table 2, the relative income position within the group—whether measured by wage income, post-transfer income, or family-supported income—did not have a significant effect on health outcomes, as indicated by the number of chronic conditions, among urban individuals. In contrast, for rural individuals, a decline in relative income position within the group was significantly associated with an increase in the number of chronic conditions, thereby indicating a deterioration in health status.

**Table 2:** Urban-rural heterogeneity of the effect of relative income position on number of multimorbidity.

**Independent Variables**	**(I)**	**(II)**	**(III)**	**(IV)**	**(V)**	**(VI)**
	**Urban**	**Rural**	**Urban**	**Rural**	**Urban**	**Rural**
*Wage income*	−0.0138 [−0.1502, 0.1227]	0.2710^***^ [0.1818, 0.3603]				
*Post-transfer*			−0.0138 [−0.1046, 0.0769]	0.1508^***^ [0.0754, 0.2262]		
*Family-supported*					0.0488 [−0.0418, 0.1395]	0.1229^***^ [0.0668, 0.1791]

Due to space limitations, lagged dependent variables and control variables are omitted; Confidence intervals are in parentheses. Variables related to absolute income level are divided by 10000. *Wage income* represents the relative position of personal wage income within the group; *Post-transfer* represents the relative position of post-transfer income within the group; *Family-supported* represents the relative position of family-supported income within the group.

This phenomenon can primarily be attributed to two factors. First, compared to urban residents, rural individuals generally have lower income levels, which restrict their access to material resources and expose them to heightened psychological stress, thus hindering their ability to prevent or treat chronic illnesses effectively. Second, there is a substantial disparity in infrastructure between urban and rural areas, including the availability of high-quality medical resources, such as tertiary hospitals, which are predominantly concentrated in urban areas. Even if urban individuals have relatively lower incomes, they still have access to medical services and living environments comparable to those of higher-income groups. While these resources may not fully match those of higher-income groups, they nevertheless play a positive role in improving health levels.

**Figure 5:**
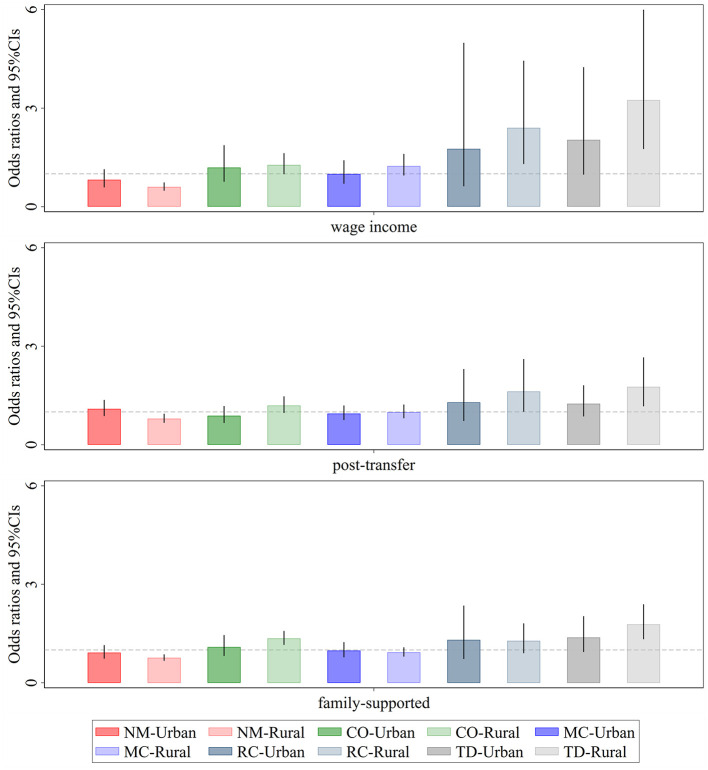
Urban-rural heterogeneity of the effect of relative income position on patterns of multimorbidity. Darker shades represent the eastern region, while lighter shades represent the non-eastern region. *Wage income* represents the relative position of personal wage income within the group; *post-transfer* represents the relative position of post-transfer income within the group; *family-supported* represents the relative position of family-supported income within the group. NM, CO, MC, RC, and TD represent No Multimorbidity, Complex-Organ diseases, Metabolic-Circulatory diseases, Respiratory-Cardiovascular diseases, and Total diseases, respectively. The coefficients display the 95% confidence intervals. Results of the panel Logit model are presented in the form of odds ratios.

From the perspective of comorbidity types, prior to income redistribution, significant urban-rural differences were observed in the effects of relative income position on Respiratory-Cardiovascular diseases and Total disease (Figure 5). The impact was more pronounced among rural individuals. However, after the interventions of government transfers and family support, the urban-rural disparity in Respiratory-Cardiovascular diseases significantly narrowed, and its prevalence became largely unrelated to relative income position. In contrast, the urban-rural disparity in Total diseases remained evident, with low-income rural individuals still facing a higher risk of severe and complex diseases. This phenomenon can be partly attributed to the fact that many rural residents, due to financial constraints, opt for “minimal treatment for major illnesses and no treatment for minor illnesses,” or even forego hospitalization altogether, leading to further deterioration of their health.

Notably, for Complex-Organ diseases, after the interventions of government transfers and family support, the prevalence among low-income rural individuals showed a significant upward trend. This finding suggests that the primary limiting factors for treating this type of comorbidity are not income level, but rather deeper underlying issues such as health beliefs, accessibility to medical resources, and the quality of healthcare services.

### 3.5 Moderating effects of developmental and health expenditures on the impact of on multimorbidity

**Figure 6:**
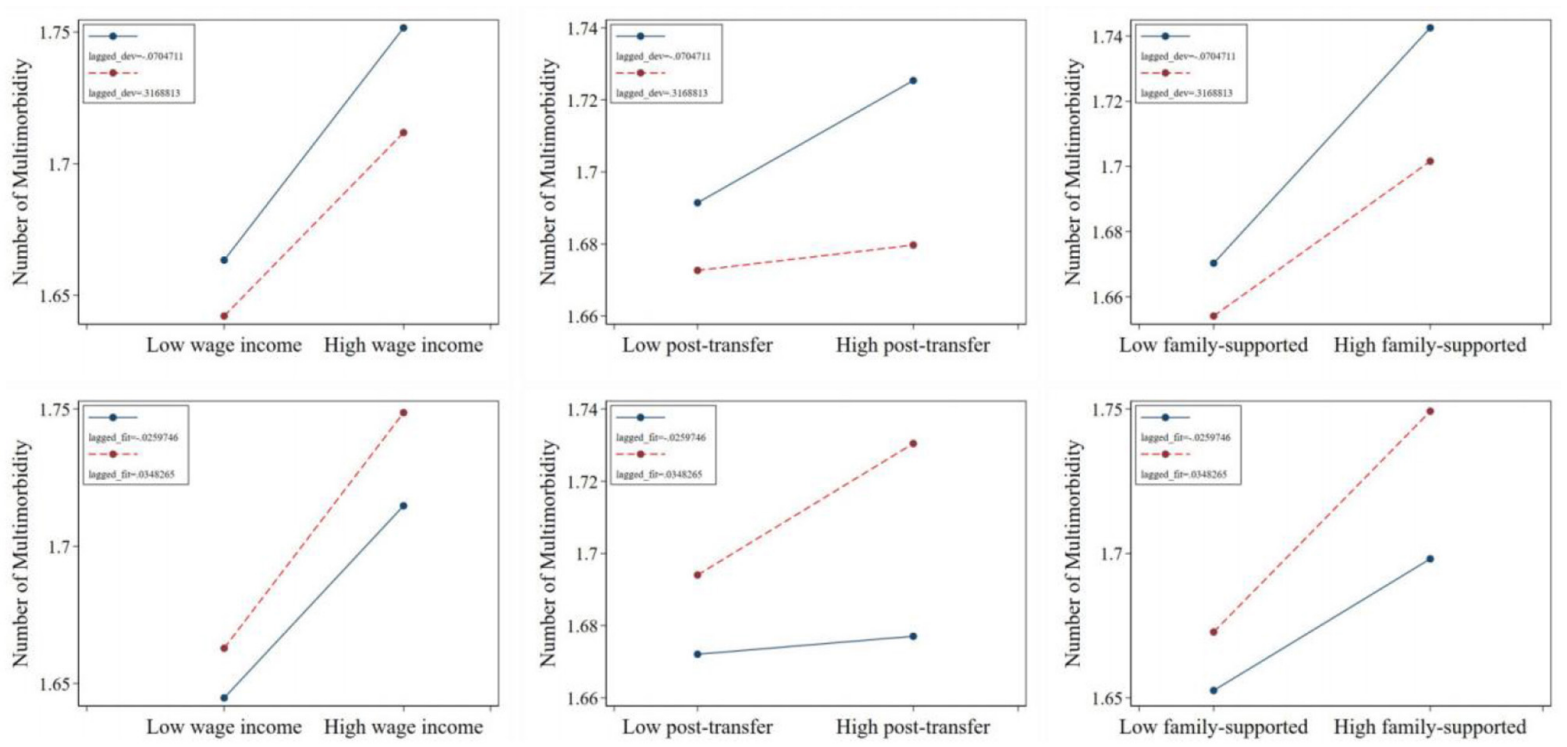
The moderating effect of related consumption decisions in the impact of relative income position on number of multimorbidity. *Wage income* represents the relative position of personal wage income within the group; *post-transfer* represents the relative position of post-transfer income within the group; *family-supported* represents the relative position of family-supported income within the group.

To explore how individuals can actively mitigate income-related health inequalities, we analyzed the moderating effects of the proportion of prior household developmental expenditures and health expenditures on the relationship between relative income position and the number of chronic diseases (Figure 6). We illustrates the relationship between the number of chronic diseases and varying Kakwani indices under different proportions of developmental and health expenditures. The average proportion of developmental (or health) expenditures, plus or minus one standard deviation, was chosen to define “high developmental (or health) expenditure proportion” and “low developmental (or health) expenditure proportion.” Similarly, high and low Kakwani indices were defined using the same method.

Regarding the moderating effect of developmental expenditures, the regression lines corresponding to a lower prior proportion of developmental expenditures consistently lie above those of higher developmental expenditures, regardless of the Kakwani index. Furthermore, the slopes for lower developmental expenditures are steeper, indicating greater sensitivity. Combined with the results from Section 3.2 and significance tests, this finding suggests that a lower proportion of developmental expenditures amplifies the promotive effect of relative transfer income position on the number of chronic diseases, leading individuals to suffer from a greater number of such conditions.

In contrast, the moderating effect of health expenditures reveals an opposite trend. The regression lines for a higher prior proportion of health expenditures consistently lie above those for lower health expenditures, and their slopes are also steeper. Combined with the results from Section 3.2 and significance tests, this indicates that a higher proportion of prior health expenditures strengthens the promotive effect of relative post-transfer income position on the number of chronic diseases. At the same time, it exacerbates the adverse effect of relative income position within family-supported contexts on the number of chronic diseases.

In addition, we also analyzed the moderating effect of the two expenditure proportions on the impact of relative income position on comorbidity types. The results, shown in Supplementary Table A.2, indicate that the moderating effect is not significant.

### 3.6 Decomposition of health inequality across various income

**Figure 7:**
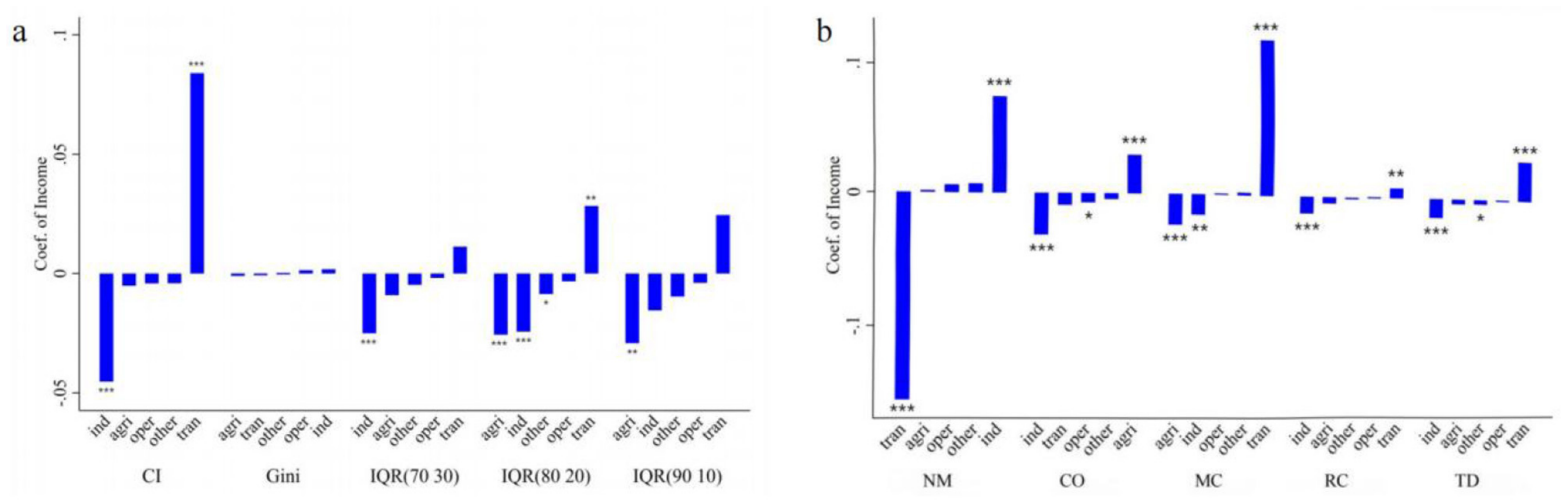
Decomposition of the inequality of multimorbidity. **(a)** Measures health inequality from the perspective of the number of chronic diseases. **(b)** Measures health inequality from the perspective of multimorbidity patterns. The height of the bars represents the magnitude of the coeffcients, the top or bottom of the bars indicates the significance of the coeffcients, and the horizontal axis represents the combinations of different measures of income and health inequality. For each dependent variable, the independent variables are arranged in ascending order based on the coefficient values. *p < 0.05, **p < 0.01, ***p < 0.001. IQR(70 30) represents the interquartile range (70th−30th percentiles) of the number of chronic diseases, IQR(80 20) represents the interquartile range (80th−20th percentiles) of the number of chronic diseases, and IQR(90 10) represents the interquartile range (90th−10th percentiles) of the number of chronic diseases. *ind* represents individual wage income, *tran* represents individual transfer income, *other* represents income from other household members, *agri* represents household agricultural operating income, and *oper* represents household non-agricultural operating income. NM, CO, MC, RC, and TD represent No Multimorbidity, Complex-Organ diseases, Metabolic-Circulatory diseases, Respiratory-Cardiovascular diseases, and Total diseases, respectively.

In terms of the number of chronic diseases, the decomposition results are shown (Figure 7a). From the perspective of the concentration index, when the average wage income of all individuals in the sample increases by one unit, the concentration index decreases by ~0.05. This indicates that an increase in average personal wage income tends to concentrate chronic diseases among the poorer population. Conversely, an increase in transfer income significantly raises the concentration index, facilitating the concentration of chronic diseases among the wealthier population. According to the Gini coefficient, different income types do not significantly impact the distribution of chronic diseases. This may be because the Gini coefficient does not reflect the distribution structure of chronic diseases within the population. From the perspective of quantile distances, an increase in average personal wage income significantly narrows the 70–30 and 80–20 quantile distances of chronic disease numbers. An increase in average transfer income widens the 80–20 quantile distance, leading to a polarization of chronic disease numbers. An increase in average income of other family members reduces the 80–20 quantile distance, slightly narrowing the gap in chronic disease numbers. Meanwhile, an increase in average family agricultural income significantly reduces the 80–20 and 90–10 quantile distances, alleviating disparities in chronic disease numbers.

In terms of multimorbidity patterns, the decomposition results are shown (Figure 7b), with four notable findings: First, an increase in average personal wage income raises the EI index of “No multimorbidity,” shifting the distribution of no chronic disease status toward the wealthier population. However, an increase in average personal transfer income shifts health status distribution toward the poorer population. Second, an increase in average personal wage income reduces the EI indices for the Complex organ, Metabolic-Circulatory, Respiratory and Cardiovascular, and total diseases patterns, concentrating these conditions among the poorer population, with the Complex organ pattern showing the greatest shift. Third, an increase in average transfer income pushes the Metabolic-Circulatory diseases toward the wealthier population, with similar effects on total diseases, Respiratory and Cardiovascular patterns, albeit to a lesser degree. However, it does not significantly affect the distribution of the Complex organ pattern. Fourth, an increase in average family agricultural income shifts the Complex organ pattern toward the wealthier population, reducing its concentration among the poor, while simultaneously concentrating Metabolic-Circulatory diseases among the poorer population.

## 4 Discussion

Firstly, China exhibits a notable shift from single chronic diseases to multimorbidity, which primarily manifests in four patterns: complex organ diseases, Metabolic-Circulatory disorders, cardio-respiratory diseases, and total diseases. Complex organ diseases are more prevalent among populations exposed to harsh environments and those with specific high-risk behaviors. Metabolic-Circulatory disorders are more strongly associated with affluent lifestyles that are nonetheless unhealthy, often referred to as “diseases of affluence.” In rapidly developing countries like China, living standards have significantly improved due to economic growth, yet health awareness has not kept pace. Overeating, physical inactivity, and high stress levels have contributed to a higher susceptibility to metabolic disorders among both impoverished and affluent groups. Respiratory and cardiovascular disorders are more common among low- to middle-income groups or in regions with significant air pollution, highly correlated with adverse environmental exposure and elevated stress levels. total diseases tends to be more prevalent among older adults, individuals living in poverty with poor lifestyle habits, populations with inadequate access to health services, or those with low adherence to treatment regimens.

Next, we examined the effect of income exploitation indices on chronic diseases from two perspectives: chronic disease prevalence and multimorbidity patterns. A lower relative position of individual wage income within a group exacerbates the burden of chronic diseases, but government redistributive policies can mitigate these adverse effects. However, even when household support is considered, having lower relative income within a group still worsens chronic disease outcomes. Therefore, income redistribution policies should strengthen income transfers that consider households as units of intervention.

Heterogeneity analysis revealed that the impact of a lower income position on the number of chronic diseases was more pronounced among individuals under the age of 65, a group that still possesses some labor capacity and can earn income either directly or indirectly through household work. However, a lower position within the income distribution may increase psychological stress, further triggering chronic diseases. Interestingly, this effect does not differ significantly between eastern and non-eastern regions. The western regions of China face unique challenges in addressing health inequalities due to their reliance on agriculture and limited healthcare infrastructure. Raising agricultural household incomes through extending agricultural value chains or promoting smart agriculture offers a double benefit—it not only improves economic well-being but also reduces the physical burden of traditional farming practices, which are often linked to chronic health conditions. For example, fostering agri-tech innovation, supporting rural e-commerce platforms, and improving access to agricultural subsidies could accelerate income growth and contribute to better health outcomes.

Regarding multimorbidity patterns, lower wage income and lower post-redistribution income are both associated with a greater likelihood of suffering from respiratory-cardiovascular diseases. However, the magnitude of this effect is greater for wage income, suggesting that income redistribution can alleviate but not eliminate this type of multimorbidity. This limitation may stem from the fact that the underlying causes of respiratory and cardiovascular disorders, such as urban air and water pollution, are forms of environmental exposure that affect both affluent and impoverished groups. This indicates that public service initiatives with broad coverage are necessary to address inequalities in respiratory-cardiovascular diseases. Furthermore, individuals disadvantaged even with household income support are likely to have limited access to social networks and medical resources, making them more susceptible to complex organ diseases or total diseases caused by adverse environmental exposures. To address this inequality, governments should prioritize providing economic support to individuals suffering from complex organ and total diseases in low-income populations.

Economic constraints also mean that relatively impoverished individuals under the age of 65 may rely more heavily on high-calorie, low-nutrition processed foods, lack a balanced diet, and, due to labor demands, lack the time or opportunity for regular exercise. These individuals may also adopt stress-coping behaviors such as smoking and excessive drinking, further exacerbating their risk for respiratory and cardiovascular disorders or total diseases (42–45). In the eastern regions, income-disadvantaged groups are more likely to consume energy-dense foods but lack adequate health awareness (46), which increases their susceptibility to Metabolic-Circulatory disorders and total diseases. In contrast, in the non-eastern regions, inadequate environmental protection and labor security make lower-income populations more likely to live and work in adverse environments, thereby significantly increasing their risk of respiratory and cardiovascular disorders. The contrasting health risks in eastern and non-eastern regions reflect the complex interplay between economic development, social inequality, and policy gaps. Addressing these disparities requires a multifaceted approach that combines health education, environmental reforms, and equitable access to medical care, tailored to the specific needs of each region.

Finally, we explored interventions to alleviate income-related chronic disease inequalities from both micro and macro perspectives. At the micro level, the effects of disadvantaged income positions on the prevalence of chronic diseases cannot be effectively mitigated through interventions such as increased investment in fitness devices or healthcare products but rely more on the improvement of living conditions, such as acquiring furniture, home appliances, and transportation equipment. These improvements address inequalities in development opportunities caused by income disparities, thereby alleviating psychological stress. Specifically, from the perspective of healthcare resource accessibility, improving living conditions, such as purchasing transportation tools, can significantly enhance access to medical resources, making it easier for patients to receive timely treatment and thereby reducing the prevalence of chronic diseases. From the perspective of fostering healthy behaviors, purchasing a refrigerator to ensure food freshness or a television to obtain health information can promote the formation of healthy habits, which in turn lowers the risk of chronic diseases. From the perspective of psychological mechanisms, purchasing furniture or household appliances can enhance living comfort and stability, thereby alleviating psychological stress and reducing the risk of chronic diseases. From the perspective of development opportunities, purchasing transportation tools (such as bicycles or electric scooters) can improve mobility, increase employment and educational opportunities, and subsequently enhance socioeconomic status, indirectly lowering the risk of chronic diseases. Although the impact is relatively modest, this still provides a potential breakthrough for policy formulation. Governments could issue subsidies for durable goods or expand scholarship programs to enhance individual development and promote health equity associated with income. The results are highly consistent with those of the regional heterogeneity analysis. In the regional heterogeneity analysis, compared to urban individuals, rural individuals face significant deficiencies in accessibility to resources such as transportation and education. This severely constrains their development opportunities, thereby increasing their risk of developing chronic diseases.

From a macro perspective, increasing average individual wage income benefits the health of affluent populations. For affluent groups, higher income allows better access to quality medical resources, healthcare services, and favorable living environments, further improving health outcomes. However, for impoverished populations, even income growth may only marginally improve health due to other factors such as limited access to healthcare resources and low health awareness. This implies that raising overall wage income levels cannot effectively address health inequalities; rather, more targeted strategies, such as income redistribution, are required. RIF regression results indicate that an increase in average individual transfer income can significantly improve the health outcomes of impoverished populations. When examining chronic disease numbers beyond income rank, increases in average wage income reduce disparities in chronic disease prevalence across the population. While there is no significant impact on the extremes (those with very few or very many chronic diseases), wage income growth appears to shift the distribution of chronic disease prevalence toward the middle. In the context of Figure 7a, this suggests that more individuals experience health deterioration while those with numerous chronic diseases decrease, reducing the disparity in chronic disease prevalence. Combined with the CI index findings, assuming chronic diseases are persistent and difficult to cure, this shift indicates that the health deterioration of certain impoverished groups contributes to the decline in disparities in chronic disease prevalence. Conversely, an increase in average individual transfer income widens disparities in chronic disease prevalence, suggesting that after income redistribution, some affluent groups with multimorbidity face greater challenges in maintaining their health. Additionally, the role of household income in influencing individual chronic disease outcomes cannot be overlooked. Raising average household agricultural income and the income of other household members can also reduce disparities in chronic disease prevalence. Particularly in China's dual-structured society (47), many individuals rely on family-based agricultural operations for income. However, agricultural income is highly uncertain, and agricultural labor poses significant health risks. Thus, increasing average agricultural income often correlates with greater labor intensity, which has a pronounced impact on health disparities, especially if the prices of basic agricultural products remain strictly regulated.

Analysis of multimorbidity patterns yields similar findings to those for chronic disease prevalence. Increasing average individual wage income benefits the health of affluent groups, a trend that is particularly evident in the case of Complex-Organ diseases. This is because Complex-Organ diseases occurs more frequently among high-risk groups exposed to harsh environments or engaging in unsafe behaviors. These groups fail to sufficiently improve their health through increased wage income due to constraints imposed by adverse living and labor conditions. While increasing average agricultural income can enable impoverished populations to escape harsh environments and working conditions, it may simultaneously make them prone to Metabolic-Circulatory disorders due to a lack of awareness about health risks associated with an improved lifestyle. Higher transfer income can improve Metabolic-Circulatory, respiratory and cardiovascular, and total diseases outcomes for impoverished groups but has limited impact on Complex-Organ diseases. This suggests the presence of immutable factors that prevent impoverished populations from alleviating complex organ diseases through increased disposable income. Therefore, relevant authorities should implement non-income-based measures, such as mandatory improvements to working environments, to mitigate this disadvantage.

There is still a lack of research on developing countries, even though many studies have explored income-related health inequalities in China—a country with distinctive characteristics, such as rapid material wealth growth but slower progress in health awareness. For example, Yao et al. utilized the EuroQol 5-Dimension-5 Level (EQ-5D-5L) to assess overall health and applied the Concentration Index (CI) decomposition method to investigate the factors driving health inequalities (14). In contrast, our study focuses on a specific health dimension—multimorbidity—to examine the factors contributing to inequalities in the distribution of chronic diseases. Tan et al. measured health levels using health-related quality of life (HRQL) and conducted a cluster analysis across 41 regions, finding that lower absolute income levels and higher intra-regional income inequality were associated with poorer health outcomes (12). This study shifts the focus to individual-level income inequality, using the Kakwani index to assess individuals' relative income positions within the sample population and offering micro-level evidence of the adverse effects of income inequality on health outcomes. Li and Tang measured health through self-rated health, chronic disease prevalence, and self-reported illnesses in the past 4 weeks. Using a non-linear decomposition of the CI index, they explored the factors influencing income-related health inequalities among residents of Western Chinese cities, finding that income explained 25–50% of health inequalities (16). While our study also employs the CI index to measure health inequality, it does not focus on income's contribution to such inequalities. Instead, using RIF regression, we investigate how increases in various types of income could mitigate health inequalities at the mean level, emphasizing the policy implications of income changes in addressing health disparities. Qin et al. also explored the relationship between chronic disease-related health inequality and income but focused only on individuals aged 45 years and older, unlike our study, which considers all age groups. They found that chronic disease prevalence was significantly associated with income levels among individuals aged 45–59. Specifically, middle-income men in this age group were more likely to develop heart disease, and middle-income women aged 45–49 were more prone to memory problems. Notably, chronic diseases in China were more prevalent among wealthier groups, differing from patterns observed in developed countries (19). Building on these findings, our study explores the relationship between income and health inequalities in the context of multimorbidity. It examines the transition from single chronic conditions to multimorbidity and differentiates various multimorbidity patterns in relation to income. Yao et al. drew on the work of Duclos et al. and argued that inequality indices measure income disparities but polarization indices better capture group clustering and social polarization, critical factors contributing to psychological stress. Their study considered health risk markers, such as BMI and blood pressure, and found that these indicators were positively associated with income polarization but displayed only weak predictive relationships with income inequality as measured by the Gini coefficient (11, 48). In contrast, our study uses the Kakwani index to provide a more precise measurement of individuals' income disadvantages and demonstrates a significant relationship with health inequalities, suggesting that income-related health inequalities may be more pronounced at the micro level.

## 5 Conclusions

This study leverages cohort data from four waves of the China Health and Retirement Longitudinal Study (CHARLS) from 2013 to 2020, utilizing Latent Class Analysis (LCA) to identify patterns of multimorbidity across chronic diseases. The Kakwani index was utilized to measure the relative position of income within groups, and panel Tobit and Logit models were applied to investigate income-related health inequalities from the perspective of chronic disease multimorbidity while examining age- and region-based heterogeneity. Furthermore, we discussed the roles of household economic decision-making and income enhancement in mitigating these health inequalities. The study yielded the following key findings:

1. China is experiencing a shift from predominantly singular chronic diseases to an increasing burden of multimorbidity. Four key multimorbidity patterns were identified: No Multimorbidity, Complex-Organ Diseases, Metabolic-Circulatory Diseases, Respiratory-Cardiovascular Diseases, and Total Diseases.
2. Lower wage income at the individual level worsens chronic disease conditions. However, government income redistribution policies can partially offset this negative impact. Even when household support for individual health is considered, low income remains a significant risk factor for chronic diseases, particularly among individuals under 65 years of age.
3. Both disadvantaged personal wage income and post-redistribution income significantly increase the likelihood of developing Respiratory-Cardiovascular diseases. Individuals with persistently low income, even with household support, are more prone to Complex-Organ diseases and total diseases. Among groups under 65, those with income disadvantages are at higher risk of Respiratory- Cardiovascular diseases and total diseases. Regionally, income-disadvantaged groups in eastern China are more likely to suffer from Metabolic-Circulatory diseases and total diseases, whereas those in non-eastern regions are more prone to Respiratory-Cardiovascular diseases.
4. Addressing the impact of income disadvantage on chronic disease prevalence proves challenging through interventions like increased exercise, medical equipment, or health supplements. Instead, it is more effectively addressed by improving living conditions. While raising average wage income benefits the health of affluent groups, addressing health inequalities requires more targeted policies. For example, increasing the redistribution of transfer income to disadvantaged populations can alleviate health inequality by improving outcomes for Metabolic-Circulatory diseases, Respiratory-Cardiovascular diseases, and total diseases, although the effect on Complex-Organ diseases is limited. Additionally, increasing household agricultural operating income may lead to a higher prevalence of Complex-Organ diseases among affluent groups but a higher prevalence of Metabolic-Circulatory diseases among disadvantaged groups.

Reducing income inequality is crucial for narrowing health disparities, but it necessitates more targeted policy measures. First, the income distribution system should be improved, with stronger redistribution efforts to effectively raise income levels for lower-income groups and enhance income transfers at the household level. Second, efforts should be made to extend agricultural industry chains and develop smart agriculture in China's non-eastern regions, increasing the added value of agricultural production to raise household agricultural operating income. Third, individual expectations for future development can be improved by offering subsidies for durable goods purchases and expanding access to scholarships. Finally, improving working environments and labor conditions, as well as enhancing the efficiency of public service provision, can provide a strong foundation for reducing health inequalities. Special attention should be given to supporting disadvantaged groups suffering from Complex-Organ diseases and total diseases.

The methodology of this study has the following limitations: First, The sample used in this study comprises over 10,000 households, covering at least 28 provinces, 150 counties, and 450 villages. However, due to data limitations, households from some provinces in China were not included in the sample. While this omission is not expected to significantly affect the results, it may limit the generalizability of the findings. Second, we did not consider the asset status of households, which could be converted into cash to support the prevention and treatment of chronic diseases, but relative data is unavailable in CHARLS. Nonetheless, as capital income is generally low among Chinese residents, this should have a minimal impact on the conclusions of this study.

## Data Availability

The original contributions presented in the study are included in the article/Supplementary material, further inquiries can be directed to the corresponding author.

## References

[1] Ignatow G Gutin I. Elite class self-interest socioeconomic inequality and US population health. Sociol Health Illn. (2024) 46:1749–71. 10.1111/1467-9566.13813 38923915

[2] Hurley J. Inequality aversion in income, health, and income-related health. J. Health Econ. (2020) 70:102276. 10.1016/j.jhealeco.2019.102276 31955864

[3] Gostin LO Friedman EA. Health Inequalities. Hast Center Rep. (2020) 50:6–8 10.2139/ssrn.3596530 PMC7267527 32356918

[4] Arcaya MC Arcaya AL Subramanian SV. Inequalities in health: definitions, concepts, and theories. Glob Health Action. (2015) 8:27106. 10.3402/gha.v8.27106 26112142 PMC4481045

[5] Harrison CA Taren D. How poverty affects diet to shape the microbiota and chronic disease. Nat Rev Immunol. (2018) 18:279–87. 10.1038/nri.2017.121 29109542

[6] Øversveen E Kelly CA. Alienation: a useful concept for health inequality research. Scand J Public Health. (2022) 50:1018–23. 10.1177/14034948221085394 35549496 PMC9578084

[7] Rod NH. The multiple layers of health inequality. Lancet Public Health. (2023) 8:e86–7. 10.1016/S2468-2667(23)00003-8 36709056

[8] Wilkinson RG Pickett KE. Income inequality and population health: a review and explanation of the evidence. Soc Sci Med. (2006) 62:1768–84. 10.1016/j.socscimed.2005.08.036 16226363

[9] Pickett KE Wilkinson RG. Income inequality and health: a causal review. Soc Sci Med. (2015) 128:316–26. 10.1016/j.socscimed.2014.12.031 25577953

[10] Adjaye-Gbewonyo K Kawachi I Subramanian SV Avendano M. Income inequality and cardiovascular disease risk factors in a highly unequal country: a fixed-effects analysis from South Africa. Int J Equity Health. (2018) 17:31. 10.1186/s12939-018-0741-0 29510733 PMC5839065

[11] Yao Y Wan G Meng D. Income distribution and health: can polarization explain health outcomes better than inequality? Eur. J Health Econ. (2019) 20:543–57. 10.1007/s10198-018-1016-9 30511340

[12] Tan Z Shi F Zhang H Li N Xu Y Liang Y. Household income, income inequality, and health-related quality of life measured by the EQ-5D in Shaanxi, China: a cross-sectional study. Int J Equity Health. (2018) 17:32. 10.1186/s12939-018-0745-9 29540183 PMC5852973

[13] Bailey HH Janssen MF Alladin FM La Foucade A Varela R Moreno JA. Evaluating health inequality in five Caribbean Basin countries using EQ-5D-5L. Appl Health Econ Health Policy. (2022) 20:857–66. 10.1007/s40258-022-00754-9 35994209 PMC9596582

[14] Yao Q Zhang X Wu Y Liu C. Decomposing income-related inequality in health-related quality of life in mainland China: a national cross-sectional study. BMJ Glob Health. (2023) 8:e013350. 10.1136/bmjgh-2023-013350 38035731 PMC10689391

[15] Costa-Font J Cowell FA Shi X. Health inequality and health insurance coverage: the United States and China compared. Econ. Hum. Biol. (2023) 52:101346. 10.2139/ssrn.4659056 38159466

[16] Li C Tang C. Income-related health inequality among rural residents in western China. Front Public Health. (2022) 10:1065808. 10.3389/fpubh.2022.1065808 36589999 PMC9797679

[17] Yang F Katumba KR Griffin S. Incorporating health inequality impact into economic evaluation in low- and middle-income countries: a systematic review. Expert Rev Pharmacoecon Outcomes Res. (2022) 22:17–25. 10.1080/14737167.2021.1954505 34263710

[18] Sturm R Gresenz CR. Relations of income inequality and family income to chronic medical conditions and mental health disorders: national survey in USA. BMJ. (2002) 324:20. 10.1136/bmj.324.7328.20 11777799 PMC61653

[19] Qin S Huang Z Ye D. Income-related inequalities in chronic disease situation among the Chinese population aged above 45 years. Inq J Health Care Organ Provis Financ. (2019) 56:0046958019860383. 10.1177/0046958019860383 31431097 PMC6704424

[20] Li H Liang H Wei L Shi D Su X Li F. Health inequality in the global burden of chronic obstructive pulmonary disease: findings from the global burden of disease study 2019. Int J Chron Obstruct Pulmon Dis Volume. (2022) 17:1695–702. 10.2147/COPD.S369120 35923358 PMC9342709

[21] He Y Zhou L Li J Wu J. An empirical analysis of the impact of income inequality and social capital on physical and mental health—take China's micro-database analysis as an example. Int J Equity Health. (2021) 20:241. 10.1186/s12939-021-01560-w 34742299 PMC8571851

[22] Bauer UE Briss PA Goodman RA Bowman BA. Prevention of chronic disease in the 21st century: elimination of the leading preventable causes of premature death and disability in the USA. Lancet. (2014) 384:45–52. 10.1016/S0140-6736(14)60648-6 24996589

[23] Niessen LW Mohan D Akuoku JK Mirelman AJ Ahmed S Koehlmoos TP. Tackling socioeconomic inequalities and non-communicable diseases in low-income and middle-income countries under the Sustainable Development agenda. Lancet. (2018) 391:2036–46. 10.1016/S0140-6736(18)30482-3 29627160

[24] Niessen LW Squire SB. Universal health coverage and chronic conditions. Lancet Glob Health. (2019) 7:e1290–2. 10.1016/S2214-109X(19)30366-3 31477546

[25] Kunna R San Sebastian M Stewart Williams J. Measurement and decomposition of socioeconomic inequality in single and multimorbidity in older adults in China and Ghana: results from the WHO study on global AGEing and adult health (SAGE). Int J Equity Health. (2017) 16:79. 10.1186/s12939-017-0578-y 28506233 PMC5433064

[26] Zhao Y Atun R Oldenburg B McPake B Tang S Mercer SW. Physical multimorbidity, health service use, and catastrophic health expenditure by socioeconomic groups in China: an analysis of population-based panel data. Lancet Glob Health. (2020) 8:e840–9. 10.1016/S2214-109X(20)30127-3 32446349 PMC7241981

[27] Mossadeghi B Caixeta R Ondarsuhu D Luciani S Hambleton IR Hennis AJM. Multimorbidity and social determinants of health in the US prior to the COVID-19 pandemic and implications for health outcomes: a cross-sectional analysis based on NHANES 2017–2018. BMC Public Health. (2023) 23:887. 10.1186/s12889-023-15768-8 37189096 PMC10183688

[28] La Porta CAM Zapperi S. Health and income inequality: a comparative analysis of USA and Italy. Front Public Health. (2024) 12:1421509. 10.3389/fpubh.2024.1421509 39171297 PMC11335724

[29] Dugravot A Fayosse A Dumurgier J Bouillon K Rayana TB Schnitzler A. Social inequalities in multimorbidity, frailty, disability, and transitions to mortality: a 24-year follow-up of the Whitehall II cohort study. Lancet Public Health. (2020) 5:e42–50. 10.1016/S2468-2667(19)30226-9 31837974 PMC7098476

[30] Buja A Claus M Perin L Rivera M Corti MC Avossa F. Multimorbidity patterns in high-need, high-cost elderly patients. PLoS ONE. (2018) 13:e0208875. 10.1371/journal.pone.0208875 30557384 PMC6296662

[31] Collins LM Lanza ST. Latent Class and Latent Transition Analysis: With Applications in the Social Behavioral, and Health Sciences, Wiley Series in Probability and Statistics (2010). Hoboken, NJ: Wiley.

[32] Kakwani N. The relative deprivation curve and its applications. J Bus Econ Stat. (1984) 2:384–94. 10.1080/07350015.1984.10509412

[33] Jiménez-Solomon O Garfinkel I Wall M Wimer C. When money and mental health problems pile up: The reciprocal relationship between income and psychological distress. SSM - Popul Health. (2024) 25:101624. 10.1016/j.ssmph.2024.101624 38380052 PMC10876910

[34] Li R Liu S Huang C Darabi D Zhao M Heinzel S. The influence of perceived stress and income on mental health in China and Germany. Heliyon. (2023) 9:e17344. 10.1016/j.heliyon.2023.e17344 37408921 PMC10318459

[35] Svalestuen S. Is the mediating effect of psychosocial stress on the income–health relationship moderated by income inequality? SSM Popul. Health. (2022) 20:101302. 10.1016/j.ssmph.2022.101302 36479320 PMC9720100

[36] Hao Y Zhang B. The impact of digital financial usage on resident's income inequality in China: An empirical analysis based on CHFS data. J Asian Econ. (2024) 91:101706. 10.1016/j.asieco.2024.101706

[37] Wu L Gao Y Niu Z Fahad S Chen R Nguyen-Thi-Lan H. A study assessing the impact of income relative deprivation and cooperative membership on rural residents' health: a pathway towards improving the health status of rural residents. One Health. (2023) 16:100494. 10.1016/j.onehlt.2023.100494 36748029 PMC9898074

[38] Zhang J Li M. Digital technology access, labor market behavior, and income inequality in rural China. Heliyon. (2024) 10:e33528. 10.1016/j.heliyon.2024.e33528 39149026 PMC11325672

[39] Zhang Y Chen C Huang L Liu G Lian T Yin M. Associations among multimorbid conditions in hospitalized middle-aged and older adults in China: statistical analysis of medical records. JMIR Public Health Surveill. (2022) 8:e38182. 10.2196/38182 36422885 PMC9732753

[40] Erreygers G. Correcting the concentration index. J Health Econ. (2009) 28:504–15. 10.1016/j.jhealeco.2008.02.003 18367273

[41] Rios-Avila F. Recentered influence functions (RIFs) in Stata: RIF regression and RIF decomposition. Stata J Promot Commun Stat Stata. (2020) 20:51–94. 10.1177/1536867X20909690

[42] Gerber Y Gabriel KP Jacobs DR Liu JY Rana JS Sternfeld B. The relationship of cardiorespiratory fitness, physical activity, and coronary artery calcification to cardiovascular disease events in CARDIA participants. Eur J Prev Cardiol. (2025) 32:52–62. 10.1093/eurjpc/zwae272 39158112 PMC11700624

[43] Santos LP. Exercise, cardiovascular health, and risk factors for atherosclerosis: a narrative review on these complex relationships and caveats of literature. Front Physiol. (2020) 11:840. 10.3389/fphys.2020.00840 32848823 PMC7411151

[44] Zahra J. Smoking, alcohol and opioids effect on coronary microcirculation: an update overview. BMC Cardiovasc Disord. (2021) 21:1–17. 10.1186/s12872-021-01990-y 33858347 PMC8051045

[45] Zampelas A Magriplis E. Dietary patterns and risk of cardiovascular diseases: a review of the evidence. Proc Nutr Soc. (2020) 79:68–75. 10.1017/S0029665119000946 31250769

[46] Briazu RA Masood F Hunt L Pettinger C Wagstaff C McCloy R. Barriers and facilitators to healthy eating in disadvantaged adults living in the UK: a scoping review. BMC Public Health. (2024) 24:1770 10.1186/s12889-024-19259-2 38961413 PMC11221142

[47] Li Y Bian Y. Social inequality in China: a review of theories and evidence. In: Social Inequality in China (London: World Scientific Publishing Europe Ltd.) (2022). p. 1–17.

[48] Duclos J-Y Esteban J Ray D. Polarization: concepts, measurement, estimation. Econometrica. (2004) 72, 1737–72. 10.1111/j.1468-0262.2004.00552.x

