# Supplementary material for: Income inequality and multimorbidity patterns in China: a micro-level analysis using CHARLS
Source: Front Public Health. 2025 Apr 16;13:1588325. doi: 10.3389/fpubh.2025.1588325 (PMC12041221; doi:10.3389/fpubh.2025.1588325)
Supplement: Supplementary file 1 [file Data_Sheet_1.pdf]

Table A.1 Characteristics of study participants

	chronic									
	0 (n=19,717)		1 (n=17,378)		2 (n=12,653)		3 (n=7,855)		>=4 (n=10,263)	
Variables	N	%	N	%	N	%	N	%	N	%
Wage income of respondents (10 hundred)										
<=0.1	14073	71.37	13198	75.95	10111	79.91	6518	82.98	8932	87.03
0.1 - 1	1431	7.26	1189	6.84	834	6.59	441	5.61	514	5.01
>1	4213	21.37	2991	17.21	1708	13.5	896	11.41	817	7.96
Transfer Income (10 hundred)										
<=0.1	16086	81.58	12755	73.4	8424	66.58	4668	59.43	5160	50.28
0.1 - 1	2194	11.13	2928	16.85	2601	20.56	1937	24.66	3098	30.19
>1	1437	7.29	1695	9.75	1628	12.87	1250	15.91	2005	19.54
Total income of other household members (10 hundred)										
<=0.1	15665	79.45	13968	80.38	10351	81.81	6591	83.91	8668	84.46
0.1 - 1	511	2.59	430	2.47	300	2.37	187	2.38	219	2.13
>1	3541	17.96	2980	17.15	2002	15.82	1077	13.71	1376	13.41
Household agricultural income (10 hundred)										
<=0	15279	77.49	12785	73.57	9143	72.26	5642	71.83	7413	72.23
0 - 0.1	748	3.79	750	4.32	599	4.73	442	5.63	572	5.57
>0.1	3690	18.71	3843	22.11	2911	23.01	1771	22.55	2278	22.2
Household operating income (10 hundred)										
<=0	17900	90.78	15975	91.93	11695	92.43	7338	93.42	9582	93.36
0 - 0.1	38	0.19	39	0.22	24	0.19	13	0.17	39	0.38
>0.1	1779	9.02	1364	7.85	934	7.38	504	6.42	642	6.26

Age										
<=45	1026	5.2	398	2.29	215	1.7	93	1.18	71	0.69
45 - 65	14638	74.24	11942	68.72	8079	63.85	4625	58.88	5337	52
>65	4053	20.56	5038	28.99	4359	34.45	3137	39.94	4855	47.31
Education										
No formal education illiterate	4140	21	4164	23.96	3044	24.06	1947	24.79	2652	25.84
Did not finish primary school but cap	3500	17.75	3300	18.99	2571	20.32	1584	20.17	2191	21.35
Sishu	48	0.24	49	0.28	37	0.29	23	0.29	25	0.24
Elementary school	4249	21.55	3875	22.3	2813	22.23	1695	21.58	2296	22.37
Middle school	4929	25	3808	21.91	2641	20.87	1594	20.29	1885	18.37
High school	1947	9.87	1378	7.93	925	7.31	617	7.85	712	6.94
Vocational school	384	1.95	392	2.26	325	2.57	218	2.78	291	2.84
Two/Three Year College / Associate de	333	1.69	269	1.55	192	1.52	119	1.51	117	1.14
Four Year College / Bachelors degree	171	0.87	131	0.75	98	0.77	54	0.69	92	0.9
Post-graduate, Masters degree	13	0.07	12	0.07	6	0.05	4	0.05	1	0.01
Post-graduate, Ph.D.	3	0.02	0	0	1	0.01	0	0	1	0.01
Self evaluation of health										
Very good	3636	18.44	1790	10.3	826	6.53	341	4.34	245	2.39
Good	3397	17.23	2233	12.85	1192	9.42	501	6.38	363	3.54
Fair	11358	57.61	10736	61.78	7684	60.73	4530	57.67	4921	47.95
Poor	1176	5.96	2186	12.58	2430	19.2	1974	25.13	3416	33.28
Very poor	150	0.76	433	2.49	521	4.12	509	6.48	1318	12.84
Alcohol										
No drinking alcohol	11903	60.37	11020	63.41	8317	65.73	5416	68.95	7517	73.24
Drink but Less Than Once A Month	1775	9	1515	8.72	1078	8.52	625	7.96	775	7.55

Drink More Than Once A Month	6039	30.63	4843	27.87	3258	25.75	1814	23.09	1971	19.2
Occupation										
Self-Employed Agricultural Work	9355	47.45	8527	49.07	5850	46.23	3325	42.33	3775	36.78
Civil servants or public institution staff	931	4.72	733	4.22	500	3.95	286	3.64	276	2.69
Company employee	1588	8.05	1116	6.42	620	4.9	306	3.9	283	2.76
Individual firm	2424	12.29	1610	9.26	965	7.63	492	6.26	472	4.6
Farmer or individual household	677	3.43	570	3.28	370	2.92	186	2.37	195	1.9
Non-agricultural self-employed work	2096	10.63	1503	8.65	974	7.7	532	6.77	569	5.54
Gender	9832	49.87	8509	48.96	5917	46.76	3568	45.42	4371	42.59
Married or cohabiting	17527	88.89	15089	86.83	10779	85.19	6546	83.34	8219	80.08
Rural household registration	15735	79.8	13881	79.88	9731	76.91	5924	75.42	7362	71.73
Smoke	6965	35.32	5688	32.73	3771	29.8	2177	27.71	2492	24.28

Table A.2 The Moderating Effect of Related Consumption Decision in the Impact of Relative Income Position on Patterns of Multimorbidity

	(I)	(II)	(III)	(IV)	(V)
	NM	CO	MC	RC	TD
wage income × lagged *de_r*	1.0371	1.1951	0.8977	0.9203	0.6441*
	[0.8407,1.2794]	[0.9308,1.5343]	[0.6966,1.1569]	[0.5235,1.6178]	[0.4158,0.9979]
post-transfer × lagged *de_r*	1.0330	1.1852	0.8725	0.8848	0.6429
	[0.8222,1.2978]	[0.9040,1.5537]	[0.6582,1.1567]	[0.4698,1.6661]	[0.3921,1.0541]
family-supported × lagged *de_r*	1.0055	1.1876	0.8695	0.9125	0.7295
	[0.7931,1.2748]	[0.8976,1.5712]	[0.6473,1.1680]	[0.4760,1.7492]	[0.4413,1.2059]
wage income × lagged *fit_r*	0.3100	1.3112	1.5008	1.6067	2.3866
	[0.0919,1.0452]	[0.2908,5.9126]	[0.4333,5.1983]	[0.0989,26.1104]	[0.4599,12.3841]

post-transfer × lagged *fit_r*	0.2423	1.1246	2.4903	2.1133	3.8094
	[0.0497,1.1805]	[0.1277,9.9037]	[0.4519,13.7243]	[0.0494,90.3287]	[0.3799,38.1982]
family-supported × lagged *fit_r*	0.2656	0.9028	2.4926	1.8359	3.6331
	[0.0561,1.2582]	[0.1026,7.9466]	[0.4712,13.1845]	[0.0441,76.3567]	[0.3852,34.2641]

Note: Due to space limitations, lagged dependent variables and control variables are omitted; * p < 0.05, ** p < 0.01, *** p < 0.001; Confidence intervals are in parentheses. *wage income* represents the relative position of personal wage income within the group; *post-transfer* represents the relative position of post-transfer income within the group; *family-supported* represents the relative position of family-supported income within the group. Results of the panel Logit model are presented in the form of odds ratios. *fit_r* represents the proportion of health expenditures in the total household expenditure; *de_r* represents the proportion of developmental expenditures in the total household expenditure.

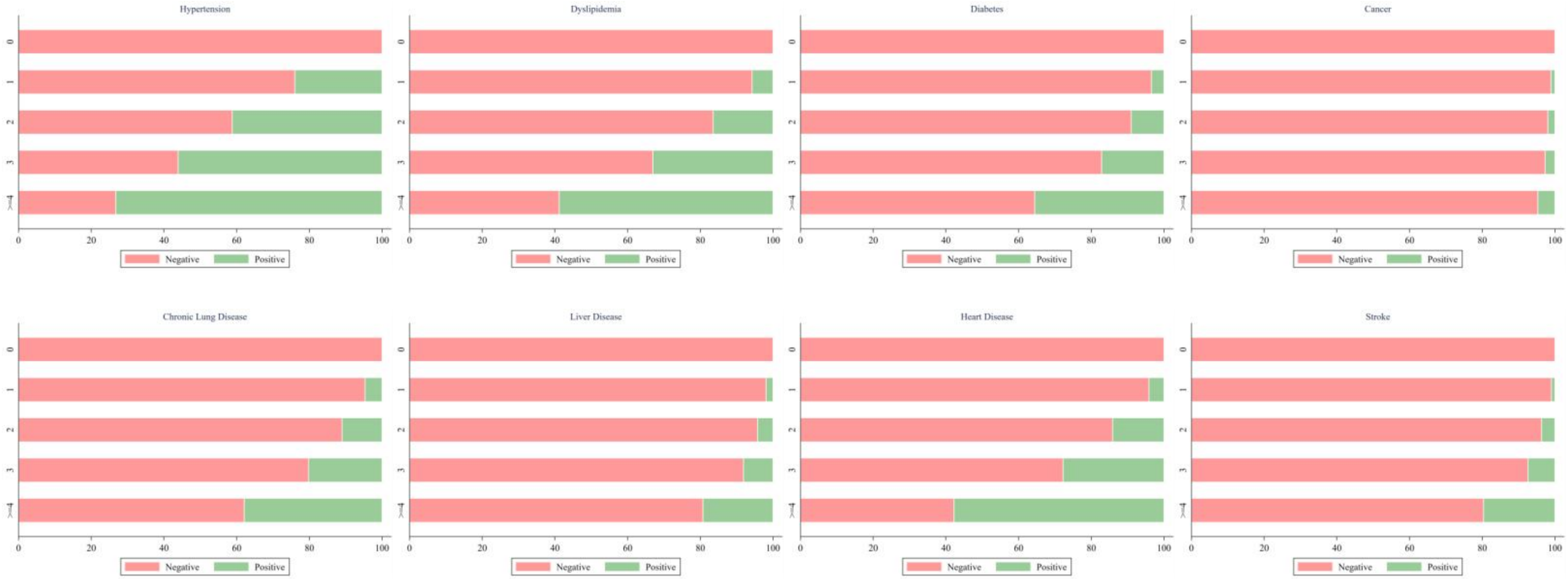

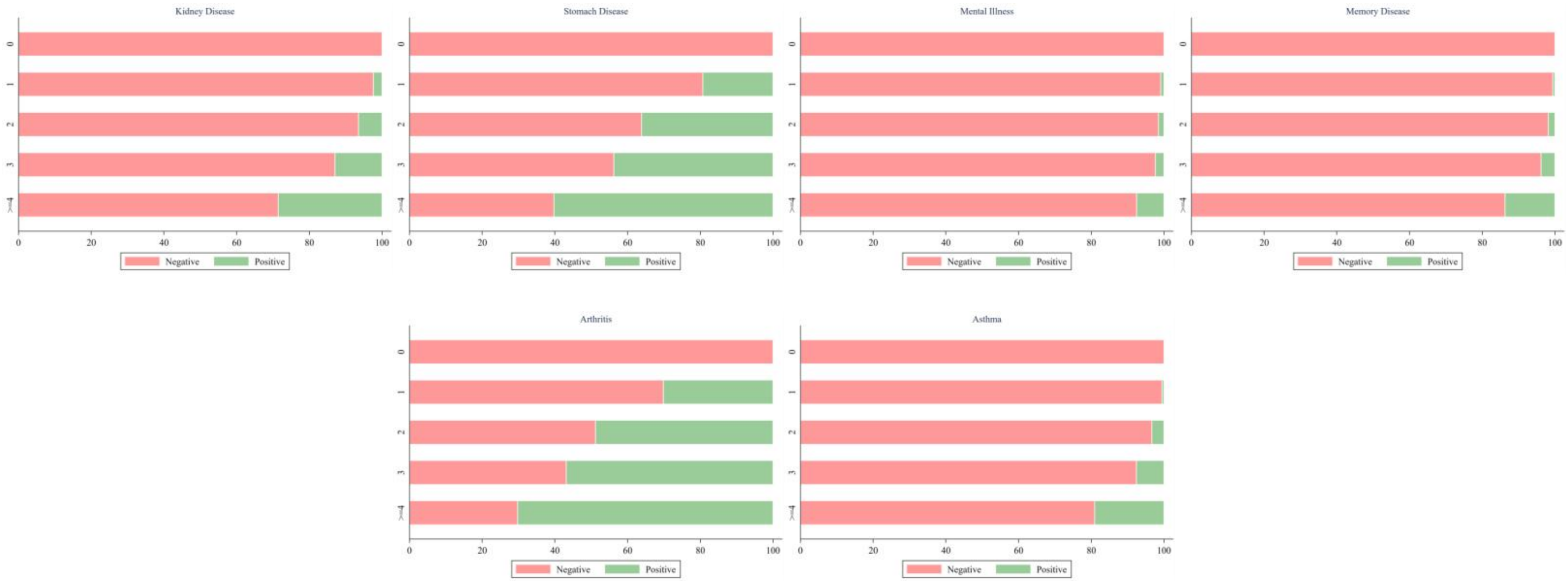

Fig.A.1 Correlation between the Number of Multimorbidity and 14 chronic diseases

Note The vertical axis shows the number of individuals with chronic diseases, while the horizontal axis represents their proportion. Red indicates the proportion of individuals without chronic diseases, while green indicates the proportion of those with chronic diseases.

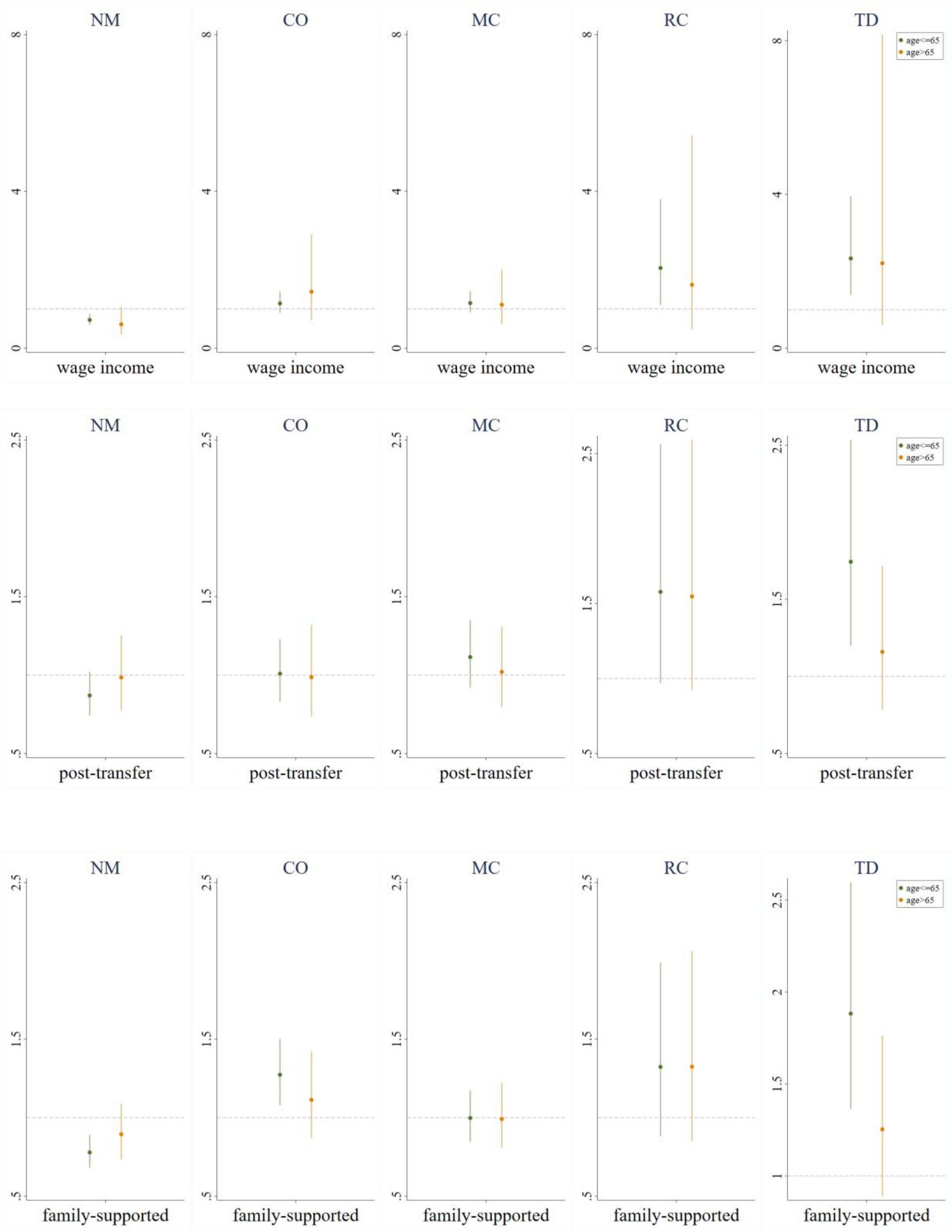

Fig.A.2 Age Heterogeneity of the Effect of Relative Income Position on Patterns of Multimorbidity

Note: *wage income* represents the relative position of personal wage income within the group; *post-transfer* represents the relative position of post-transfer income within the group; *family-supported* represents the relative position of family-supported income within the group. NM, CO, MC, RC and TD represent No Multimorbidity, Complex-Organ diseases, Metabolic-Circulatory diseases, Respiratory-Cardiovascular diseases and Total diseases, respectively. Results of the panel Logit model are presented in the form of odds ratios. The coefficients are presented with 95% confidence intervals
